# Supplementary material for: The insect somatostatin pathway gates vitellogenesis progression during reproductive maturation and the post-mating response
Source: Nat Commun. 2022 Feb 18;13:969. doi: 10.1038/s41467-022-28592-2 (PMC8857180; doi:10.1038/s41467-022-28592-2)
Supplement: Supplementary file 1 — Supplementary Information [file 41467_2022_28592_MOESM1_ESM.pdf]

The insect somatostatin pathway gates vitellogenesis progression during reproductive maturation and the post-mating response

Zhang et al.

Supplementary Information

Supplementary Figures and Legends

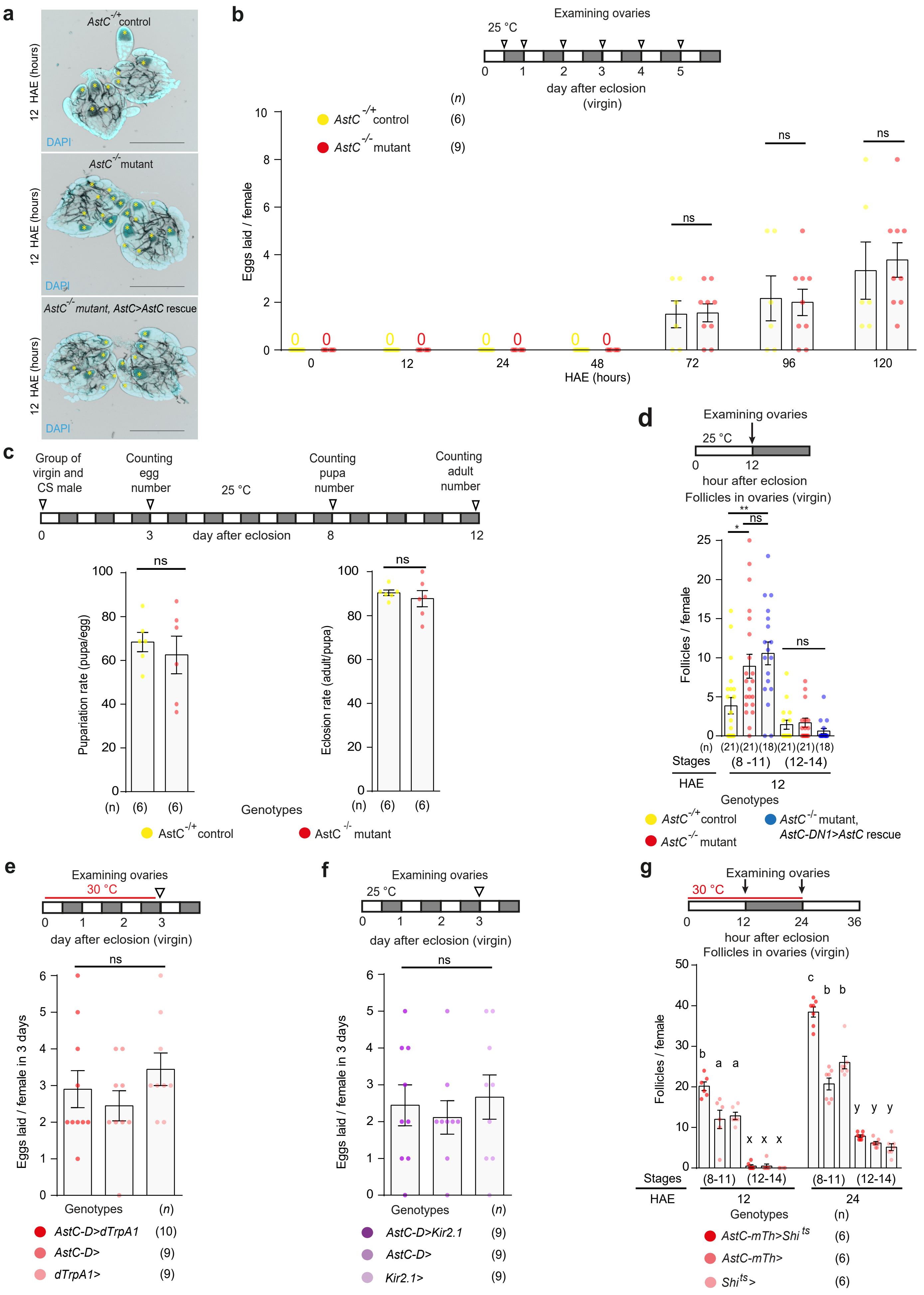

Supplementary Fig. 1 AstC neuropeptide or *AstC-mTh* neurons do not alter egg-laying activity in virgin females, and AstC-DN1 is not involved in vitellogenesis progression during reproductive maturation. **(a)** Confocal Z-projection images of ovaries stained with DAPI (blue) from indicated genotypes 12 hours after eclosion (HAE). The ovary images were acquired with infrared and DAPI channels. Yellow asterisks indicate oocytes in early vitellogenetic stages (stages 8-11). Scale bar, 500 μm. **(b)** Above, experimental protocol. Below, number of eggs laid per virgin female of the indicated genotypes at the indicated hours after eclosion (HAE) under 25°C. Two-tailed unpaired *t* test; ns (non-significance), $p > 0.05$. $p=0.93$ (72 HAE), $p=0.87$ (96 HAE), $p= 0.74$ (120 HAE). Error bars indicate s.e.m. **(c)** Above, experimental protocol. Below, pupariation rate (left) and eclosion rate (right) of the indicated genotypes maintained at 25°C. Two-tailed unpaired *t* test, ns (non-significance), $p > 0.05$. $p=0.55$ (pupariation rate), $p=0.51$ (eclosion rate). Error bars represent s.e.m. **(d)** Above, experimental protocol. Below, number of stage 8–11 and 12–14 follicles per virgin female of the indicated genotype 12 hours after eclosion (HAE) maintained at 25°. One-way ANOVA followed by Tukey's test for multiple comparisons; *$p < 0.05$; **$p < 0.01$; ns (non-significance), $p > 0.05$. Error bars represent s.e.m. **(e, f)** Above, experimental protocol. Below, number of eggs laid per virgin female of the indicated genotype during 3 days after eclosion maintained at 30°C or 25°C. One-way ANOVA followed by Tukey's test for multiple comparisons; ns (non-significance), $p > 0.05$. Error bars represent s.e.m. **(g)** Above, experimental protocol. Below, number of stage 8–11 and 12–14 follicles per virgin female of the indicated genotype at the indicated number of hours after eclosion (HAE) while maintained at 30°. The letters above the columns indicate significant differences ($p < 0.05$) for comparisons of time points and genotypes (two-way ANOVA with Bonferroni post-hoc test for multiple comparisons); a-c for stage 8–11 and xy for stage 12–14. Error bars represent s.e.m. For a summary of statistical analyses including adjusted *p* values and a detailed list of genotypes, see Supplementary Tables 1-2, respectively.

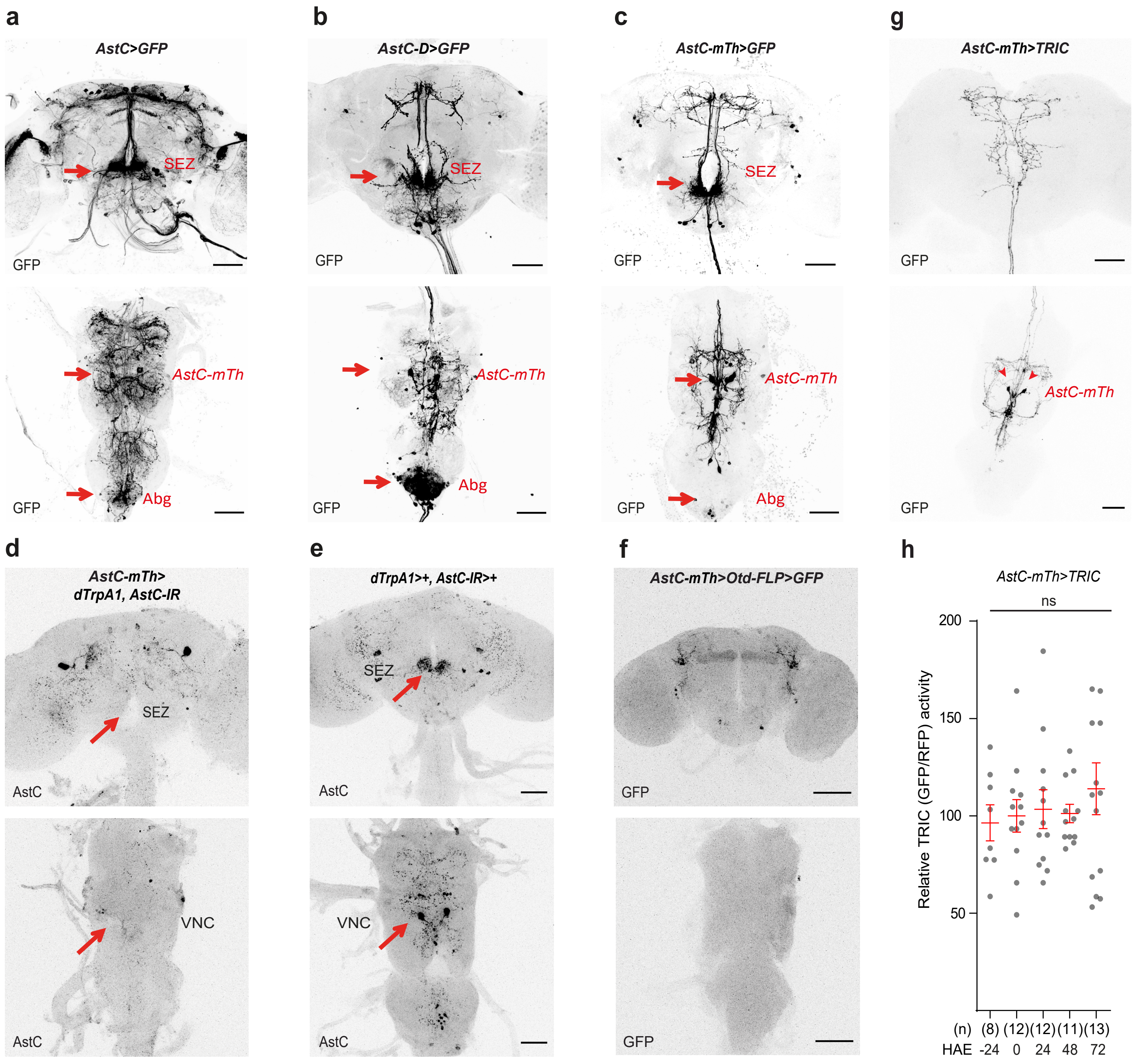

Supplementary Fig. 2 AstC-mTh neurons and their Ca2+ activity during reproductive maturation. (a, b,and c) Confocal Z-projection images of the brain (above) and VNC (below) of 4-day-old virgin females carrying *UAS-mCD8-EGFP* and *AstC-Gal4* (a), *AstC-D-Gal4* (b), or *AstC-mTh-Gal4* (c) stained with anti-GFP (black). Arrows indicate the mesothorasic ganglion, the SEZ in the brain, and the abdominal ganglion. Scale bars, 50 μm. **(d, e)** Negative images of the brain (above) and VNC (below) from 4-day-old virgin females of the indicated genotypes stained with anti-AstC (black). Arrows indicate the SEZ and VNC regions in which *AstC-RNAi* under the control of *AstC-mTh-Gal4* led to an attenuation of anti-AstC labeling. Note that the presence of *AstC-RNAi* resulted in a concomitant loss of anti-AstC labeling in the SEZ and *AstC-mTh* neuron somas. Scale bars, 50 μm. **(f)** Negative images of the brain (above) and VNC (below) from 4-day-old virgin females carrying *AstC-mTh-Gal4, Otd^{Flp}, UAS>stop>UAS-GFP* stained with anti-GFP. Scale bars, 50 μm. **(g)** Negative images of TRIC labelling (anti-GFP) of pairs of *AstC-mTh* neurons in the brain (above) and VNC (below) of 4-day-old virgin females. Labeling indicates intracellular Ca^{2+} transients. TRIC expressed under the control of *AstC-mTh-Gal4* labeled one of two *AstC-mTh* neurons. Arrowheads (red) indicate *AstC-mTh* neuron somas. Scale bars, 50 μm. **(h)** The GFP intensity in *AstC-mTh* neurons of TRIC females shows Ca^{2+} activity in virgin females at the indicated hours after eclosion (HAE). One-way ANOVA followed by Tukey's test for multiple comparisons; *ns* (non-significance), $p > 0.05$. Error bars indicate s.e.m. For a summary of statistical analyses including adjusted *p* values and a detailed list of genotypes, see Supplementary Tables 1-2, respectively.

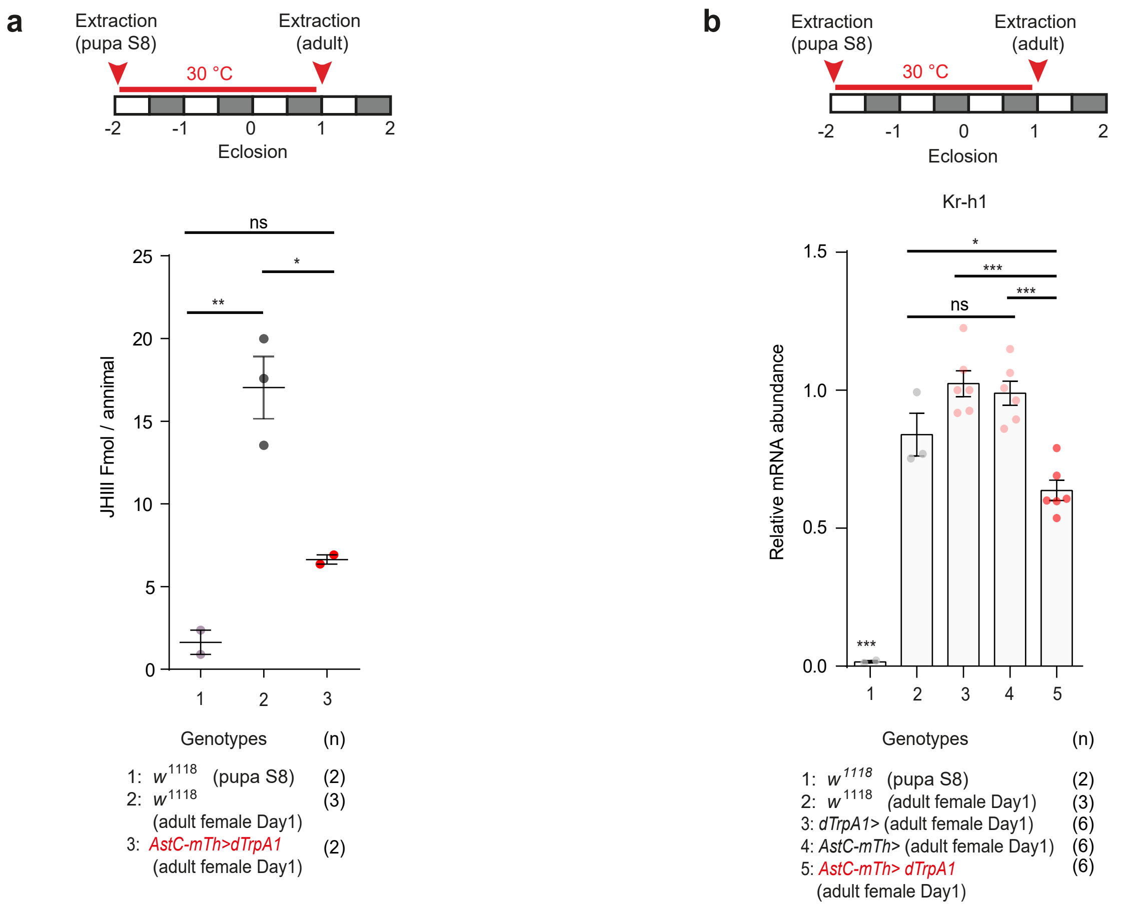

Supplementary Fig. 3 Activation of *AstC-mTh-Gal4* neurons reduces JH-III biosynthesis. **(a)** Above, experimental protocol for thermal activation followed by JH-III analysis. Below, JH-III levels from whole body extracts of *w^1118^* S8 stage pupae (2 days before eclosion, day -2), *w^1118^* adult females (1 day after eclosion, day 1), and *AstC-mTh-Gal4, UAS-dTrpA1* adult females (day 1). Each circle indicates the average JH-III level from 10 females. One-way ANOVA followed by Tukey's test for multiple comparisons. *$p < 0.05$; **$p < 0.01$; ns (non-significance), $p > 0.05$. Error bars represent s.e.m. **(b)** Above, experimental protocol. Below, *Kr-h1* transcript levels in S8 stage pupae and adult females of the indicated genotypes maintained for 3 days at 30°C. One-way ANOVA followed by Tukey's test for multiple comparisons. *$p < 0.05$; ***$p < 0.001$; ns (non-significance), $p > 0.05$. Error bars indicate s.e.m. Note that all control genotypes (#2–4) show similar levels of *Kr-h1* expression, whereas the test group (genotype #5) that showed marked reduction in JHIII also showed a ~40% reduction in *Kr-h1* expression. Genotypes #3-5 was shown in Fig 3a. For a summary of statistical analyses including adjusted *p* values and a detailed list of genotypes, see Supplementary Tables 1-2, respectively.

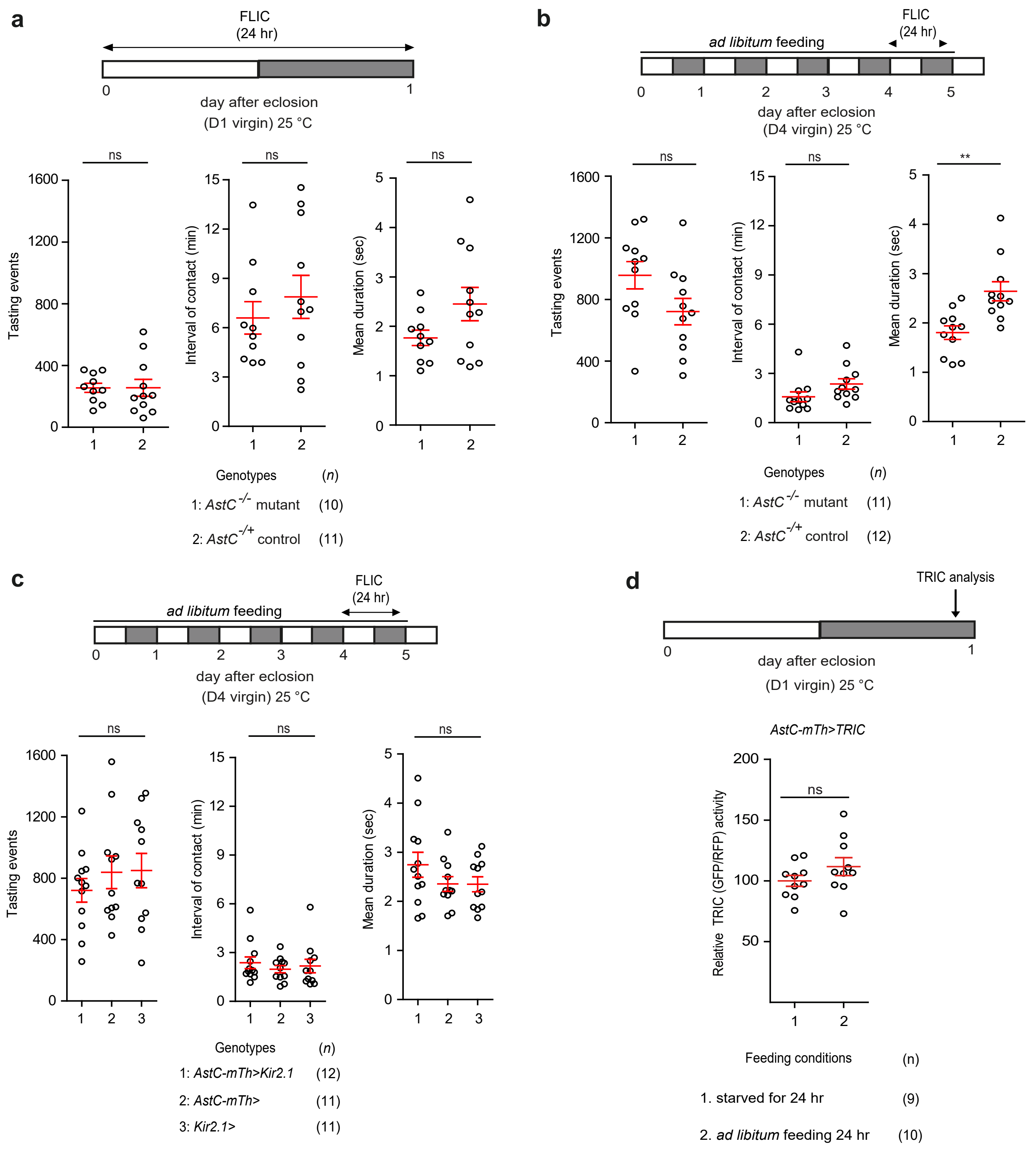

Supplementary Fig. 4 *AstC-mTh* neurons have a limited impact on feeding. **(a-c)** Above, experimental protocol for the FLIC system. Below, feeding parameters monitored in virgin females of the indicated genotype using the FLIC system. Each circle indicates individual flies examined. Two-tailed unpaired *t* test **(a, b)**, $^{**}p < 0.01$; ns (non-significance), $p > 0.05$. p=0.99, p=0.45, p=0.09 (Tasting, interval, mean duration in **a**); p=0.07, p=0.09, p=0.002 (Tasting, interval, mean duration in **b**). Error bars represent s.e.m. One-way ANOVA followed by Tukey's test for multiple comparisons among genotypes **(c),** ns (non-significance), $p > 0.05$. Error bars represent s.e.m. (**d**) Above, experimental protocol. Below, relative TRIC (GFP/RFP) intensities in *AstC-mTh* neurons of TRIC females showing Ca^{2+} activity in virgin females subjected to the indicated feeding condition. Two-tailed unpaired *t* test; *ns* (non-significance), $p > 0.05$, p= 0.19. Error bars indicate s.e.m. For a summary of statistical analyses including adjusted *p* values and a detailed list of genotypes, see Supplementary Tables 1-2, respectively.

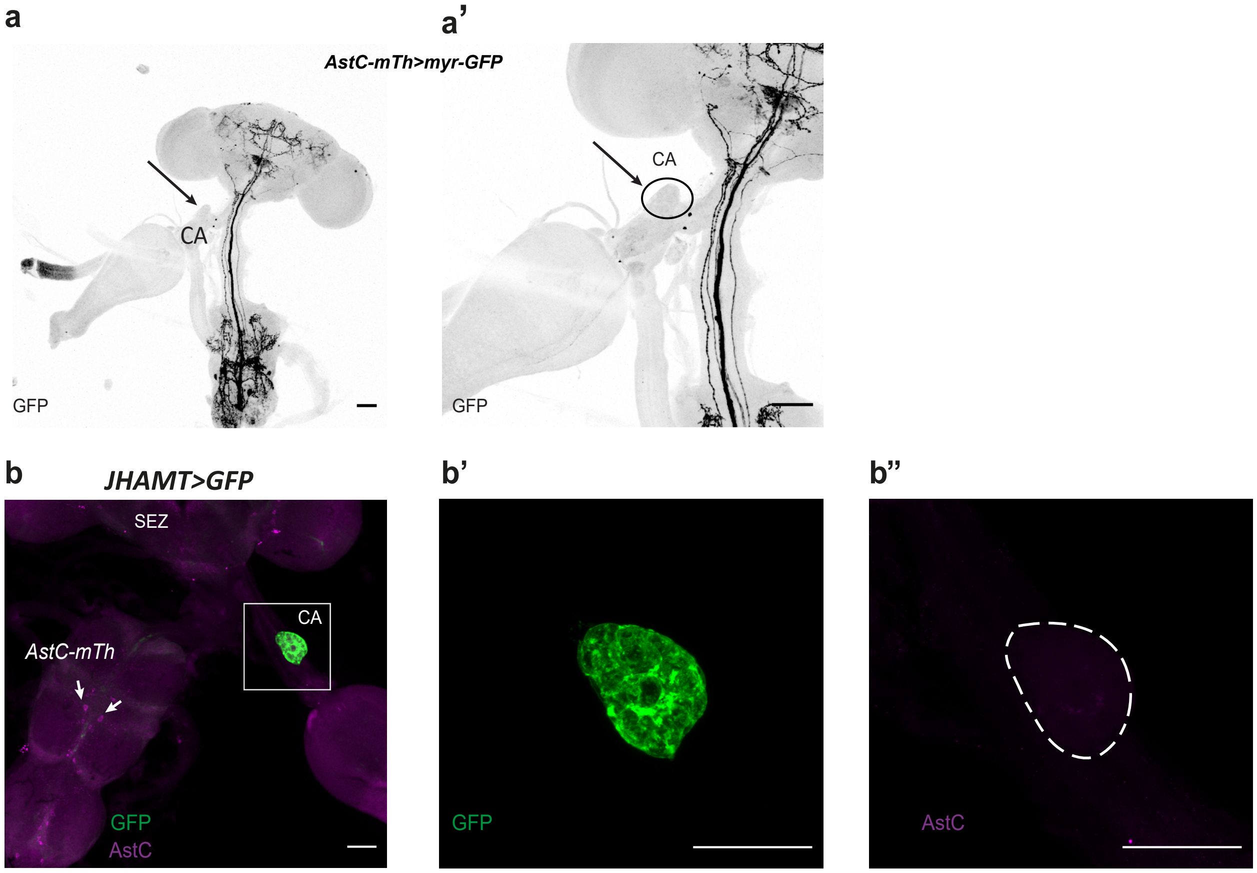

Supplementary Fig. 5 *AstC-mTh* neurons do not innervate the CA. **(a)** Negative image of the brain-CA complex of 4-day-old virgin females of the indicated genotype stained with anti-GFP (Black). The black arrows indicate the CA. (**a’)** represents a higher magnification image of **(a)**. Scale bars, 50 µm. (**b)** Confocal Z-projection images of the brain-CA complex of 4-day-old virgin females carrying *JHAMT Gal4* and *UAS-myr-GFP* stained with anti-AstC (magenta) and anti-GFP (green). (**b’** and **b”)** are higher magnifications of the inset box (white) in (**b)**. Scale bars, 50 µm. For a detailed list of genotypes, see Supplementary Table 2.

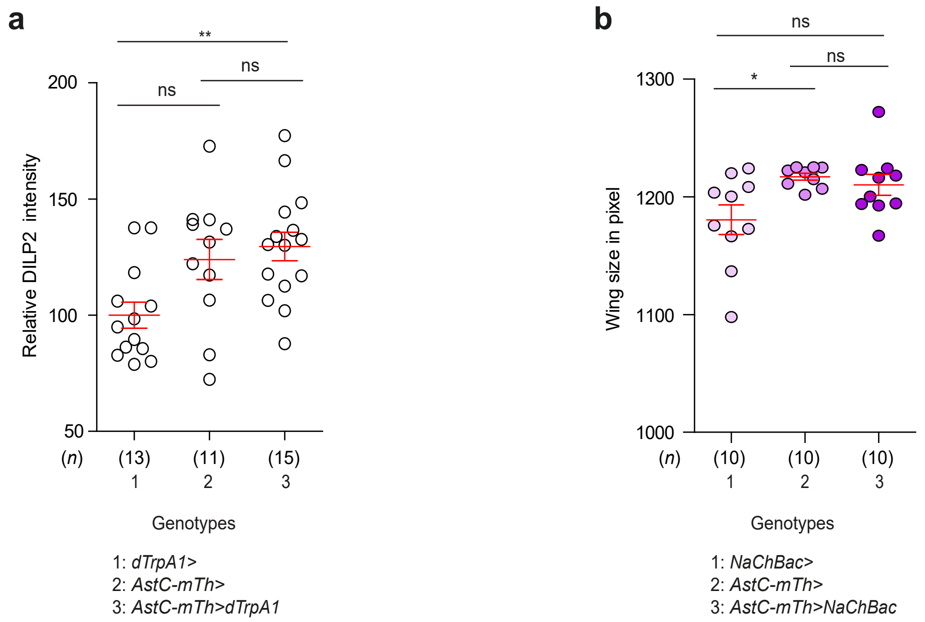

Supplementary Fig. 6 *AstC-mTh* neurons have a limited influence on insulin signaling. **(a)** Relative fluorescence intensity of anti-Dilp2 staining of the brain insulin-producing cells from virgin females of the indicated genotype maintained for 3 days post-eclosion at 30°C. Each circle indicates the relative fluorescence intensity of all the IPC somas from a single brain. The relative fluorescence intensity was calculated by setting the average of the control group (genotype 1, *dTrpa1>*) to 100%. One-way ANOVA followed by Tukey's test for multiple comparisons **(a, b)**; **$p < 0.01$; *$p < 0.05$; *ns* (non-significance) $p > 0.05$. Error bars represent s.e.m. **(b)** Wing size in pixels as measured for the indicated genotypes. 1 pixel is close to 2.5 μm. Error bars indicate s.e.m. For a summary of statistical analyses including adjusted *p* values and a detailed list of genotypes, see Supplementary Tables 1-2, respectively.

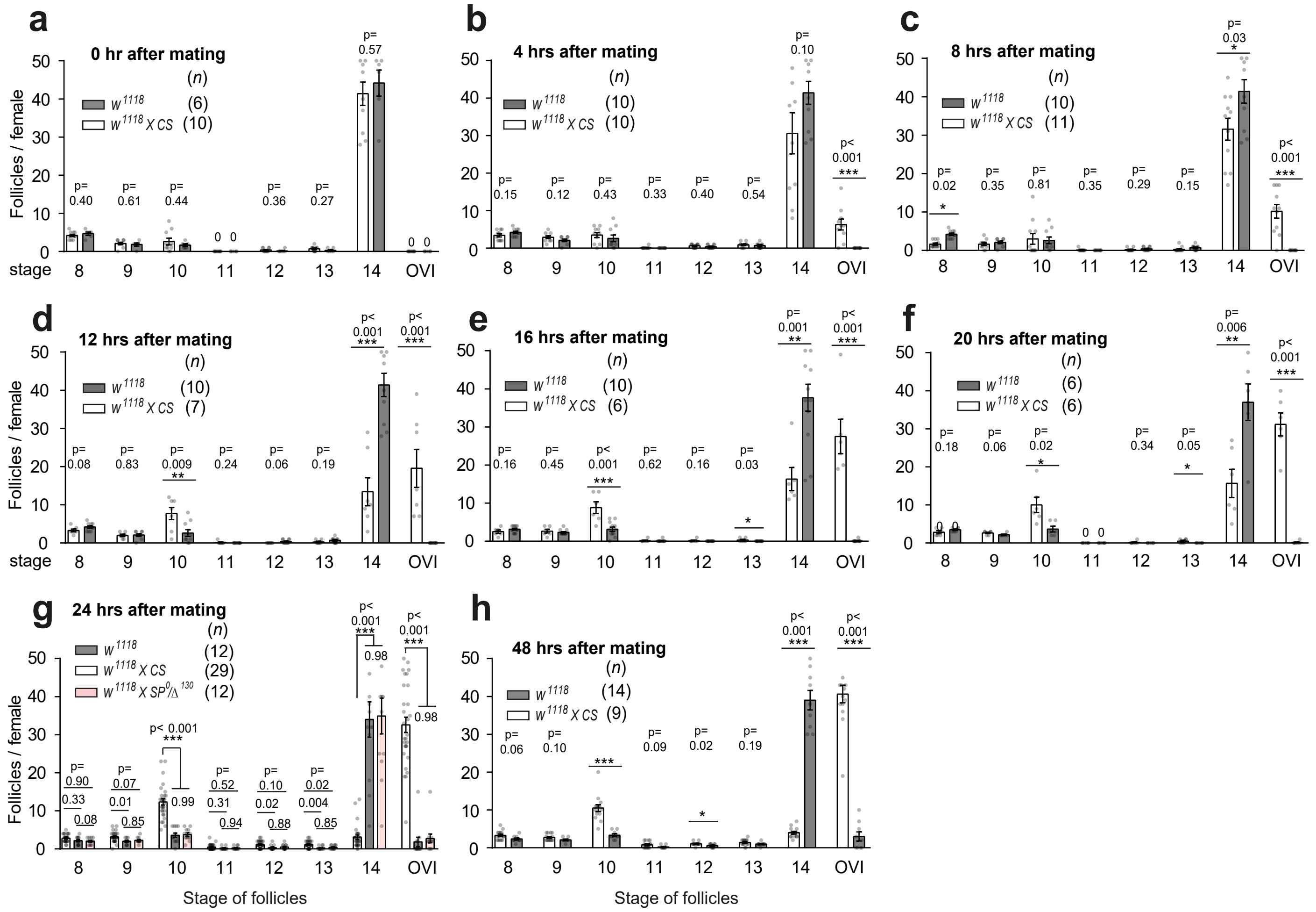

Supplementary Fig. 7 Mating-induced vitellogenesis in *Drosophila melanogaster.* The number of stage 8–14 vitellogenic follicles and oviposited eggs (OVI) from w^{1118} virgin females (gray bars) or females mated with wild-type *Canton-S* (*CS*) males (white bars) or SP-less males (SP^0/Δ^{130}) (pink bar in g) at the indicated hours after mating. Virgin females were age-matched. Two-tailed unpaired *t* tests **(a-f, h)**; One-way ANOVA followed by Tukey's test for multiple comparisons **(g)**; ***$p < 0.001$; **$p < 0.01$; *$p < 0.05$; no labeling, $p > 0.05$. Error bars indicate s.e.m. For a detailed list of genotypes, see Supplementary Table 2.

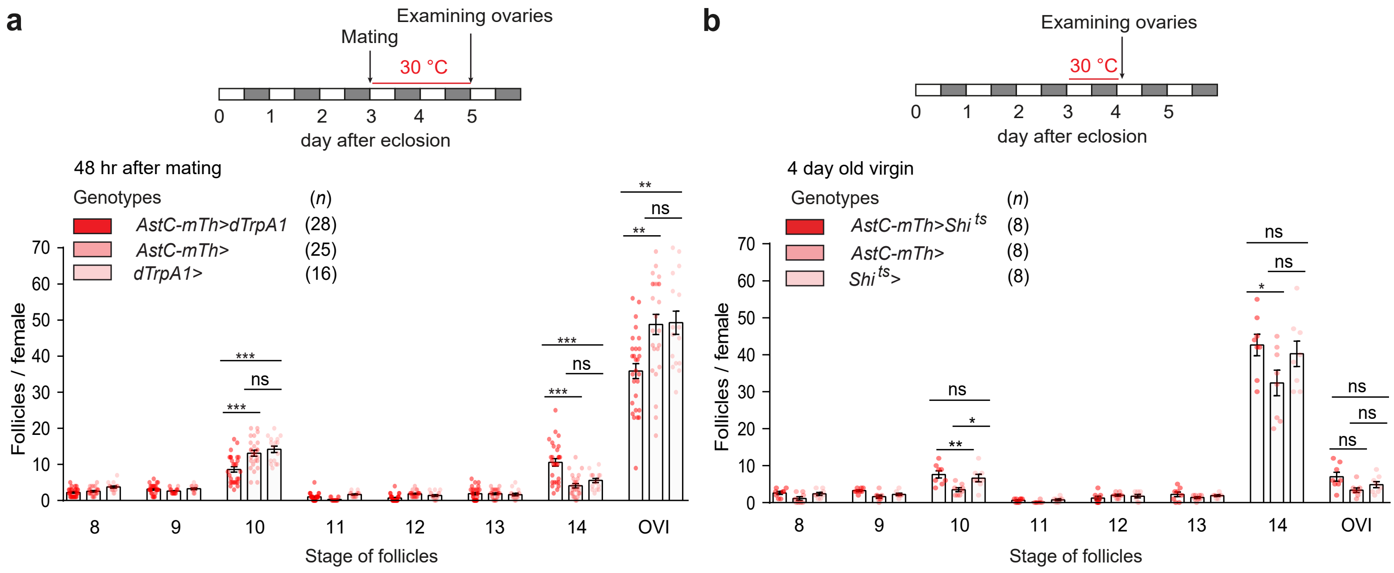

Supplementary Fig. 8 *AstC-mTh* neurons gate post-mating vitellogenesis. **(a and b)** Above, experimental protocol. Below, number of follicles of the indicated stage and oviposited eggs (OVI) from females of the indicated genotype 48 hours after mating and temperature shift **(a)** or 24 hours after temperature shift **(b)**. One-way ANOVA followed by Tukey's test for multiple comparisons among genotypes; ***$p < 0.001$; **$p < 0.01$; *$p < 0.05$; ns (non-significance) and no labeling, $p > 0.05$. Error bars indicate s.e.m. For a summary of statistical analyses including adjusted p values and a detailed list of genotypes, see Supplementary Tables 1-2, respectively.

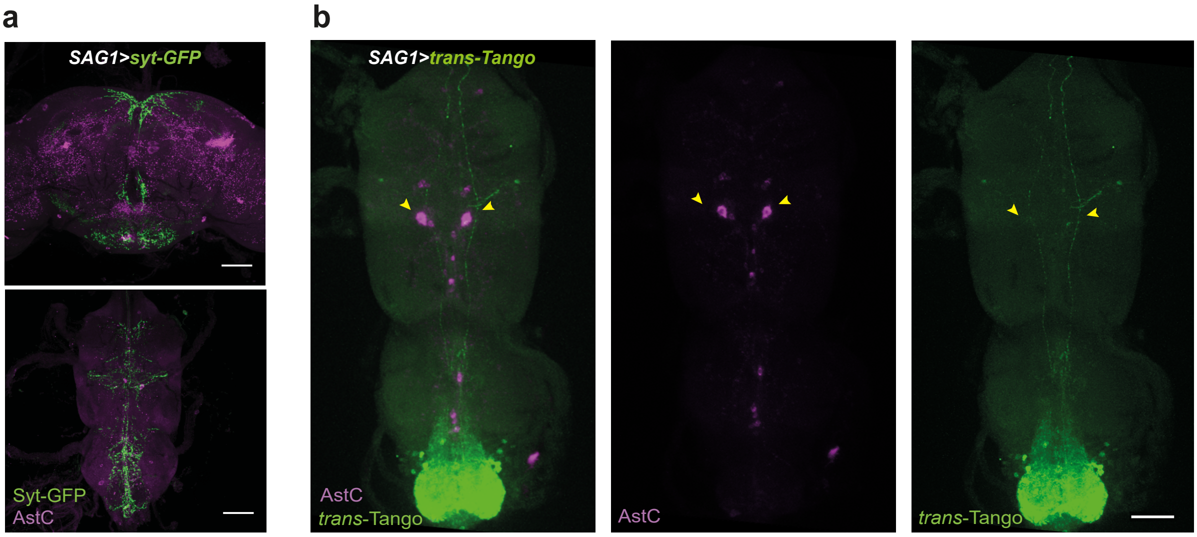

Supplementary Fig. 9 Anatomical interactions between SAG neurons and *AstC-mTh* neurons. **(a)** Confocal Z-projection images of the brain (above) and VNC (below) of 4-day-old virgin females carrying *SAG1-Gal4* and *UAS-syt-GFP* stained with anti-GFP. Scale bars, 50 μm. (**b)** Confocal Z-projection images of the VNC of 4-day-old virgin females carrying *SAG1-Gal4* and *trans-Tango* stained with anti-AstC (magenta) and anti-HA (*trans*-Tango, green). The trans-Tango signal (green) does not colocalize with *AstC-mTh* neurons (arrows). Scale bar, 50 μm. For a detailed list of genotypes, see Supplementary Table 2.

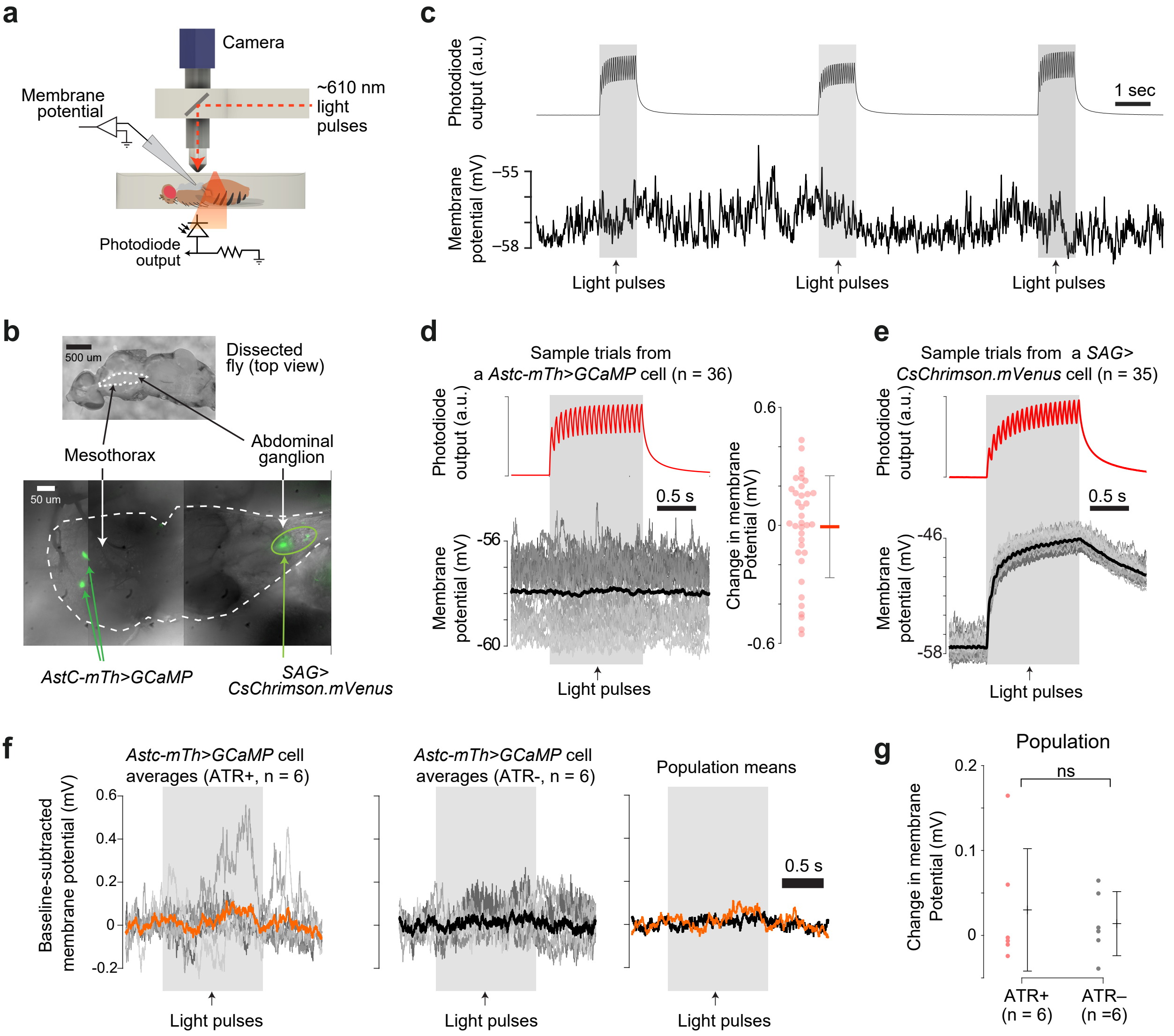

Supplementary Fig. 10 Optogenetic activation of SAG neurons failed to induce a significant change in the membrane potential of *AstC-mTh* neurons. **(a)** Experimental setup (not to scale). **(b)** Dissected images of a sample fly (top) and its VNC (bottom). The VNC images for infrared and GFP channels were acquired separately and overlaid post hoc. Scale bars are indicated. **(c)** Photodiode output (top) and membrane potential from a sample *AstC-mTh* neuron (3-day-old female, retinal-fed and mated). **(d)** *AstC-mTh* neuron in a fly fed with all-trans-retinal (ATR+) were aligned to the onset of light pulses. Thick red (top) and black(bottom) lines indicate the mean of the trials. Individual trials were plotted in gray. Error bars indicate s.d. **(e)** Individual trials of a sample *SAG>CsChrimson* neuron (ATR-fed) in response to the light stimulation. A thick black line and a red line indicate the means of individual trials. **(f)** Average traces for a population of ATR+ flies and non-retinal-fed (ATR–) flies. The mean traces were plotted on the right for comparison. **(g)** The mean change in membrane in the membrane potential for the ATR+ and ATR- flies. Each dot represents the mean change in membrane potential for a cell, measured by subtracting the mean baseline amplitude (500-ms interval immediately preceding the light pulse onset) from the mean membrane potential within the light pulse interval (1.2-s interval after the light pulse onset). The two-tailed unpaired *t* test was used for analysis. ns (non-significance) and no labeling, $p > 0.05$. Error bars indicate s.d. For detailed list of genotypes, see Supplementary Table 2.

Supplementary Table 1. A summary of statistical analyses used in this study.

Figure	Statistical method		
Fig.1a	Two-way ANOVA followed by Bonferroni post tests		
	AstC1/+,AstC-Gal4/+ (8-11)	Summary	Adjusted P Value
	0 vs. 12 HAE	ns	0.22
	0 vs. 24 HAE	***	<0.001
	0 vs. 48 HAE	***	<0.001
	0 vs. 72 HAE	*	0.04
	0 vs. 96 HAE	**	0.005
	0 vs. 120 HAE	***	<0.001
	12 vs. 24 HAE	***	<0.001
	12 vs. 48 HAE	***	<0.001
	12 vs. 72 HAE	ns	>0.99
	12 vs. 96 HAE	ns	>0.99
	12 vs. 120 HAE	***	<0.001
	24 vs. 48 HAE	ns	>0.99
	24 vs. 72 HAE	***	<0.001
	24 vs. 96 HAE	***	<0.001
	24 vs. 120 HAE	ns	0.15
	48 vs. 72 HAE	***	<0.001
	48 vs. 96 HAE	***	<0.001
	48 vs. 120 HAE	**	0.004
	72 vs. 96 HAE	ns	>0.99
	72 vs. 120 HAE	ns	0.07
	96 vs. 120 HAE	ns	0.09
	AstC1/AstC1 (8-11)		
	0 vs. 12 HAE	***	<0.001
	0 vs. 24 HAE	***	<0.001
	0 vs. 48 HAE	***	<0.001
	0 vs. 72 HAE	***	<0.001
	0 vs. 96 HAE	**	0.002
	0 vs. 120 HAE	***	<0.001
	12 vs. 24 HAE	***	<0.001
	12 vs. 48 HAE	*	0.02
	12 vs. 72 HAE	ns	>0.99
	12 vs. 96 HAE	*	0.04
	12 vs. 120 HAE	ns	>0.99
	24 vs. 48 HAE	***	<0.001
	24 vs. 72 HAE	***	<0.001
	24 vs. 96 HAE	***	<0.001
	24 vs. 120 HAE	***	<0.001
	48 vs. 72 HAE	*	0.01
	48 vs. 96 HAE	***	<0.001
	48 vs. 120 HAE	ns	0.24
	72 vs. 96 HAE	ns	>0.99
	72 vs. 120 HAE	ns	>0.99
	96 vs. 120 HAE	*	0.01
	AstC1/AstC1,AstC-Gal4/UAS-AstC (8-11)		
	0 vs. 12 HAE	ns	0.08
	0 vs. 24 HAE	***	<0.001
	0 vs. 48 HAE	***	<0.001
	0 vs. 72 HAE	***	<0.001
	0 vs. 96 HAE	***	<0.001
	0 vs. 120 HAE	***	<0.001
	12 vs. 24 HAE	***	<0.001
	12 vs. 48 HAE	***	<0.001
	12 vs. 72 HAE	ns	>0.99
	12 vs. 96 HAE	ns	0.43
	12 vs. 120 HAE	ns	0.05
	24 vs. 48 HAE	ns	0.18
	24 vs. 72 HAE	***	<0.001
	24 vs. 96 HAE	**	0.006
	24 vs. 120 HAE	ns	0.9
	48 vs. 72 HAE	***	<0.001
	48 vs. 96 HAE	***	<0.001
	48 vs. 120 HAE	***	<0.001
	72 vs. 96 HAE	ns	>0.99
	72 vs. 120 HAE	ns	>0.99
	96 vs. 120 HAE	ns	>0.99
	AstC1/+,AstC-Gal4/+ (12-14)	Summary	Adjusted P Value
	0 vs. 12 HAE	ns	>0.99
	0 vs. 24 HAE	ns	0.81
	0 vs. 48 HAE	***	<0.001
	0 vs. 72 HAE	***	<0.001
	0 vs. 96 HAE	***	<0.001
	0 vs. 120 HAE	***	<0.001
	12 vs. 24 HAE	ns	0.73
	12 vs. 48 HAE	***	<0.001
	12 vs. 72 HAE	***	<0.001
	12 vs. 96 HAE	***	<0.001
	12 vs. 120 HAE	***	<0.001
	24 vs. 48 HAE	***	<0.001
	24 vs. 72 HAE	***	<0.001
	24 vs. 96 HAE	***	<0.001
	24 vs. 120 HAE	***	<0.001

Figure	Statistical method		
Fig. 1c	One-way ANOVA followed by Tukey's multiple comparison test		
		Summary	Adjusted P Value
	AstC-A>TrpA1 vs. AstC-A>	ns	>0.99
	AstC-A>TrpA1 vs. AstC-B>TrpA1	ns	>0.99
	AstC-A>TrpA1 vs. AstC-B>	ns	>0.99
	AstC-A>TrpA1 vs. AstC-C>TrpA1	ns	>0.99
	AstC-A>TrpA1 vs. AstC-C>	ns	>0.99
	AstC-A>TrpA vs. AstC-D>TrpA	***	<0.001
	AstC-A>TrpA1 vs. AstC-D>	ns	0.61
	AstC-A>TrpA1 vs. AstC-E>TrpA1	ns	0.92
	AstC-A>TrpA1 vs. AstC-E>	ns	>0.99
	AstC-A>TrpA1 vs. Trpa1>	ns	>0.99
	AstC-A> vs. AstC-B>TrpA1	ns	>0.99
	AstC-A> vs. AstC-B>W	ns	>0.99
	AstC-A> vs. AstC-C>TrpA1	ns	>0.99
	AstC-A> vs. AstC-C>	ns	0.99
	AstC-A> vs. AstC-D>TrpA1	***	<0.001
	AstC-A> vs. AstC-D>	ns	0.08
	AstC-A> vs. AstC-E>TrpA1	ns	0.36
	AstC-A> vs. AstC-E>	ns	0.85
	AstC-A> vs. +Trpa1>	ns	0.96
	AstC-B>TrpA1 vs. AstC-B>	ns	>0.99
	AstC-B>TrpA1 vs. AstC-C>TrpA1	ns	>0.99
	AstC-B>TrpA1vs. AstC-C>	ns	>0.99
	AstC-B>TrpA1 vs. AstC-D>TrpA1	***	<0.001
	AstC-B>TrpA1 vs. AstC-D>	ns	0.59
	AstC-B>TrpA1 vs. AstC-E>TrpA1	ns	0.91
	AstC-B>TrpA1 vs. AstC-E>	ns	>0.99
	AstC-B>TrpA1 vs. Trpa1>	ns	>0.99
	AstC-B> vs. AstC-C>TrpA1	ns	>0.99
	AstC-B> vs. AstC-C>	ns	>0.99
	AstC-B> vs. AstC-D>TrpA1	***	<0.001
	AstC-B> vs. AstC-D>	ns	0.28
	AstC-B> vs. AstC-E>TrpA1	ns	0.72
	AstC-B> vs. AstC-E>	ns	>0.99
	AstC-B> vs. Trpa1>	ns	>0.99
	AstC-C>TrpA1 vs. AstC-C>	ns	>0.99
	AstC-C>TrpA1 vs. AstC-D>TrpA1	***	<0.001
	AstC-C>TrpA1vs. AstC-D>	ns	0.13
	AstC-C>TrpA1 vs. AstC-E>TrpA1	ns	0.43
	AstC-C>TrpA1 vs. AstC-E>	ns	0.89
	AstC-C>TrpA1 vs.Trpa1>	ns	0.97
	AstC-C> vs. AstC-D>TrpA1	***	<0.001
	AstC-C> vs. AstC-D>	ns	0.58
	AstC-C> vs. AstC-E>TrpA1	ns	0.92
	AstC-C> vs. AstC-E>	ns	>0.99
	AstC-C> vs. Trpa1>	ns	>0.99
	AstC-D>TrpA1 vs. AstC-D>	***	<0.001
	AstC-D>TrpA1 vs. AstC-E>TrpA1	***	<0.001
	AstC-D>TrpA1 vs. AstC-E>	***	<0.001
	AstC-D>TrpA1 vs. Trpa1>	***	<0.001
	AstC-D> vs. AstC-E>TrpA1	ns	>0.99
	AstC-D> vs. AstC-E>	ns	0.96
	AstC-D> vs. Trpa1>	ns	0.84
	AstC-E>TrpA1 vs. AstC-E>	ns	>0.99
	AstC-E>TrpA1 vs.Trpa1>	ns	0.99
	AstC-E> vs. Trpa1>	ns	>0.99
Fig. 1d	One-way ANOVA followed by Tukey's multiple comparison test		
		Summary	Adjusted P Value
	AstC1>+, AstC-D>Trpa1 vs. AstC1/AstC1, AstC-D>Trpa1	***	<0.001
	AstC1>+, AstC-D>Trpa1 vs. AstC1>+	***	<0.001
	AstC1>+, AstC-D>Trpa1 vs. AstC1/AstC1	***	<0.001
	AstC1/AstC1, AstC-D>Trpa1 vs. AstC1>+	ns	0.11
	AstC1/AstC1, AstC-D>Trpa1 vs. AstC1>AstC1	*	0.01
	AstC1>AstC1 vs. AstC1>+	ns	0.78
Fig. 1e	Two-way ANOVA followed by Bonferroni post tests		
	12 HAE	Summary	Adjusted P Value
	AstC-D>Kir2.1 (8-11) vs AstC-D-Gal4> (8-11)	***	<0.001
	AstC-D>Kir2.1 (8-11) vs. Kir2.1> (8-11)	***	<0.001
	AstC-D> (8-11) vs. Kir2.1> (8-11)	ns	>0.99
	24 HAE		
	AstC-D>Kir2.1 (8-11) vs AstC-D-Gal4> (8-11)	**	0.001
	AstC-D>Kir2.1 (8-11) vs. Kir2.1> (8-11)	***	<0.001
	AstC-D> (8-11) vs. Kir2.1> (8-11)	ns	0.74
	12 HAE		
	AstC-D>Kir2.1 (12-14) vs AstC-D-Gal4> 12-14)	ns	>0.99
	AstC-D>Kir2.1 12-14) vs. Kir2.1> 12-14)	ns	>0.99
	AstC-D> 12-14) vs. Kir2.1> 12-14)	ns	>0.99
	24 HAE		
	AstC-D>Kir2.1 (12-14) vs AstC-D-Gal4> 12-14)	ns	>0.99
	AstC-D>Kir2.1 12-14) vs. Kir2.1> 12-14)	ns	>0.99

48 vs. 72 HAE	***	<0.001
48 vs. 96 HAE	***	<0.001
48 vs. 120 HAE	***	<0.001
72 vs. 96 HAE	ns	0.49
72 vs. 120 HAE	**	0.001
96 vs. 120 HAE	ns	0.75
AstC1/AstC1 (12-14)		
0 vs. 12 HAE	ns	>0.99
0 vs. 24 HAE	ns	0.18
0 vs. 48 HAE	***	<0.001
0 vs. 72 HAE	***	<0.001
0 vs. 96 HAE	***	<0.001
0 vs. 120 HAE	***	<0.001
12 vs. 24 HAE	ns	0.12
12 vs. 48 HAE	***	<0.001
12 vs. 72 HAE	***	<0.001
12 vs. 96 HAE	***	<0.001
12 vs. 120 HAE	***	<0.001
24 vs. 48 HAE	***	<0.001
24 vs. 72 HAE	***	<0.001
24 vs. 96 HAE	***	<0.001
24 vs. 120 HAE	***	<0.001
48 vs. 72 HAE	***	<0.001
48 vs. 96 HAE	***	<0.001
48 vs. 120 HAE	***	<0.001
72 vs. 96 HAE	*	0.01
72 vs. 120 HAE	**	0.002
96 vs. 120 HAE	ns	>0.99
AstC1/AstC1,AstC-Gal4/UAS-AstC (12-14)		
0 vs. 12 HAE	ns	>0.99
0 vs. 24 HAE	ns	0.18
0 vs. 48 HAE	***	<0.001
0 vs. 72 HAE	***	<0.001
0 vs. 96 HAE	***	<0.001
0 vs. 120 HAE	***	<0.001
12 vs. 24 HAE	ns	0.27
12 vs. 48 HAE	***	<0.001
12 vs. 72 HAE	***	<0.001
12 vs. 96 HAE	***	<0.001
12 vs. 120 HAE	***	<0.001
24 vs. 48 HAE	***	<0.001
24 vs. 72 HAE	***	<0.001
24 vs. 96 HAE	***	<0.001
24 vs. 120 HAE	***	<0.001
48 vs. 72 HAE	***	<0.001
48 vs. 96 HAE	***	<0.001
48 vs. 120 HAE	***	<0.001
72 vs. 96 HAE	ns	0.65
72 vs. 120 HAE	***	<0.001
96 vs. 120 HAE	**	0.004
Two-way ANOVA followed by Bonferroni post tests		
	Summary	Adjusted P Value
0 HAE		
AstC1/+,AstC-Gal4/+ (8-11) vs. *AstC1/AstC1* (8-11)	ns	>0.99
AstC1/+,AstC-Gal4/+ (8-11) vs. *AstC1/AstC1>AstC/AstC* (8-11)	ns	>0.99
AstC1/AstC1 (8-11) vs. *AstC1/AstC1,AstC-Gal4/UAS-AstC* (8-11)	ns	>0.99
12 HAE		
AstC1/+,AstC-Gal4/+ (8-11) vs. *AstC1/AstC1* (8-11)	***	<0.001
AstC1/+,AstC-Gal4/+ (8-11) vs. *AstC1/AstC1>AstC/AstC* (8-11)	ns	>0.99
AstC1/AstC1 (8-11) vs. *AstC1/AstC1,AstC-Gal4/UAS-AstC* (8-11)	***	<0.001
24 HAE		
AstC1/+,AstC-Gal4/+ (8-11) vs. *AstC1/AstC1* (8-11)	***	<0.001
AstC1/+,AstC-Gal4/+ (8-11) vs. *AstC1/AstC1>AstC/AstC* (8-11)	*	0.03

	AstC-D> 12-14) *vs. Kir2.1>* 12-14)	ns	>0.99
	Two-way ANOVA followed by Bonferroni post tests		
	12 -24 HAE	Summary	Adjusted P Value
	AstC-D>Kir2.1 (8-11)	***	<0.001
	AstC-D> (8-11)	***	<0.001
	Kir2.1> (8-11)	***	<0.001
	12 - 24 HAE		
	AstC-D>Kir2.1 (12-14)	***	<0.001
	AstC-D> (12-14)	***	<0.001
	Kir2.1> (12-14)	***	<0.001

Fig. 1f	One-way ANOVA followed by Tukey's multiple comparison test		
		Summary	Adjusted P Value
	AstC1/+,AstC-Gal4/+ (8-11) vs. *AstC1/AstC1* (8-11)	***	<0.001
	AstC1/+,AstC-Gal4/+ (8-11) vs. *AstC1/AstC1>AstC/-DAstC* (8-11)	*	0.03
	AstC1/AstC1 (8-11) vs. *AstC1/AstC1,AstC-Gal4/UAS-AstC* (8-11)	***	<0.001
	AstC1/+,AstC-Gal4/+ (12-14) vs. *AstC1/AstC1* (12-14)	ns	0.96
	AstC1/+,AstC-Gal4/+ (12-14) vs. *AstC1/AstC1>AstC/-DAstC* (12-14)	ns	0.34
	AstC1/AstC1 (12-14) vs. *AstC1/AstC1,AstC-Gal4/UAS-AstC* (12-14)	ns	0.51

Fig. 2a	One-way ANOVA followed by Tukey's multiple comparison test		
		Summary	Adjusted P Value
	Dicer2; AstC-D split >Trpa1 vs. AstC-D split >Trpa1 AstC-IR	***	<0.001
	Dicer2; AstC-D split >Trpa1 vs. *Dicer2; AstC-D split >*	***	<0.001
	Dicer2; AstC-D split >Trpa1 vs. *Trpa1 AstC-IR>*	***	<0.001
	Dicer2; AstC-D split >Trpa1 vs. Trpa1>	***	<0.001
	AstC-D split >Trpa1 AstC-IR vs. Dicer2; AstC-D split >	ns	0.79
	AstC-D split >Trpa1 AstC-IR vs. *Trpa1 AstC-IR >*	ns	0.37
	AstC-D split >Trpa1 AstC-IR vs. *Trpa1>*	ns	>0.99
	Dicer2; AstC-D split >vs.Trpa1 AstC-IR>	*	0.03
	Dicer2; AstC-D split > vs. *Trpa1>*	ns	0.74
	Trpa1 AstC-IR> vs. *Trpa1>*	ns	0.76

Fig. 2d	One-way ANOVA followed by Tukey's multiple comparison test		
		Summary	Adjusted P Value
	AstC-D split >otd-flp Trpa1 vs. AstC-D split >Trpa1	***	<0.001
	AstC-D split >otd-flp Trpa1 vs. AstC-D split>	ns	0.92
	AstC-D split >otd-flp Trpa1 vs. otd-flp Trpa1>	ns	0.97
	AstC-D split >otd-flp Trpa1 vs. Trpa1>	ns	>0.99
	AstC-D split >Trpa1 vs. AstC-D split>	***	<0.001
	AstC-D split >Trpa1 vs. otd-flp Trpa1>	***	<0.001
	AstC-D split >Trpa1 vs. Trpa1>	***	<0.001
	AstC-D split> vs. otd-flp Trpa1>	ns	>0.99
	AstC-D split> vs. Trpa1>	ns	0.7
	otd-flp Trpa1> vs. Trpa1>	ns	0.82

Fig. 3a	One-way ANOVA followed by Tukey's multiple comparison test		
		Summary	Adjusted P Value
	Trpa1> D1 adult vs. *AstC-D split >* D1 adult	ns	0.84
	Trpa1> D1 adult vs. *AstC-D split >Trpa1* D1 adult	***	<0.001
	AstC-D split > D1 adult vs. *AstC-D split >Trpa1* D1 adult	***	<0.001

Fig. 3b	One-way ANOVA followed by Tukey's multiple comparison test		
		Summary	Adjusted P Value
	AstC-D split >TrpA1 vs. AstC-D split >TrpA1 (M)	***	<0.001
	AstC-D split >TrpA1 vs. AstC-D split >	***	<0.001
	AstC-D split >TrpA1 vs. Trpa1>	***	<0.001
	AstC-D split >TrpA1 (M) vs. AstC-D split >	ns	0.52
	AstC-D split >TrpA1 (M) vs. Trpa1>	***	<0.001
	AstC-D split > vs.Trpa1>	*	0.03

Fig. 3e	Two-way ANOVA followed by Bonferroni post tests		
	12 HAE	Summary	Adjusted P Value

	AstC1/AstC1 (8-11) vs. AstC1/AstC1,AstC-Gal4/UAS-AstC (8-11)	***	<0.001
	48 HAE		
	AstC1/+,AstC-Gal4/+ (8-11) vs. AstC1/AstC1 (8-11)	ns	0.15
	AstC1/+,AstC-Gal4/+ (8-11) vs. AstC1/AstC1>AstC/AstC (8-11)	ns	0.77
	AstC1/AstC1 (8-11) vs. AstC1/AstC1,AstC-Gal4/UAS-AstC (8-11)	ns	>0.99
	72 HAE		
	AstC1/+,AstC-Gal4/+ (8-11) vs. AstC1/AstC1 (8-11)	ns	0.35
	AstC1/+,AstC-Gal4/+ (8-11) vs. AstC01/AstC01>AstC/AstC (8-11)	ns	>0.99
	AstC1/AstC1 (8-11) vs. AstC1/AstC1,AstC-Gal4/UAS-AstC (8-11)	ns	0.48
	96 HAE		
	AstC1/+,AstC-Gal4/+ (8-11) vs. AstC1/AstC1 (8-11)	ns	>0.99
	AstC1/+,AstC-Gal4/+ (8-11) vs. AstC1/AstC1>AstC/AstC (8-11)	ns	>0.99
	AstC1/AstC1 (8-11) vs. AstC1/AstC1,AstC-Gal4/UAS-AstC (8-11)	ns	>0.99
	120 HAE		
	AstC1/+,AstC-Gal4/+ (8-11) vs. AstC1/AstC1 (8-11)	ns	>0.99
	AstC1/+,AstC-Gal4/+ (8-11) vs. AstC1/AstC1>AstC/AstC (8-11)	ns	0.56
	AstC1/AstC1 (8-11) vs. AstC1/AstC1,AstC-Gal4/UAS-AstC (8-11)	ns	0.55
	0 HAE		
	AstC1/+,AstC-Gal4/+ (12-14) vs. AstC1/AstC1 (12-14)	ns	>0.99
	AstC1/+,AstC-Gal4/+ (12-14) vs. AstC1/AstC1>AstC/AstC (12-14)	ns	>0.99
	AstC1/AstC1 (12-14) vs. AstC1/AstC1,AstC-Gal4/UAS-AstC (12-14)	ns	>0.99
	12 HAE		
	AstC1/+,AstC-Gal4/+ (12-14) vs. AstC1/AstC1 (12-14)	ns	>0.99
	AstC1/+,AstC-Gal4/+ (12-14) vs. AstC01/AstC01>AstC/AstC (12-14)	ns	>0.99
	AstC1/AstC1 (12-14) vs. AstC1/AstC1,AstC-Gal4/UAS-AstC (12-14)	ns	>0.99
	24 HAE		
	AstC1/+,AstC-Gal4/+ (12-14) vs. AstC1/AstC1 (12-14)	ns	>0.99
	AstC1/+,AstC-Gal4/+ (12-14) vs. AstC1/AstC1>AstC/AstC (12-14)	ns	>0.99
	AstC1/AstC1 (12-14) vs. AstC1/AstC1,AstC-Gal4/UAS-AstC (12-14)	ns	>0.99
	48 HAE		
	AstC1/+,AstC-Gal4/+ (12-14) vs. AstC1/AstC1 (12-14)	*	0.04
	AstC1/+,AstC-Gal4/+ (12-14) vs. AstC1/AstC1>AstC/AstC (12-14)	ns	>0.99
	AstC1/AstC1 (12-14) vs. AstC1/AstC1,AstC-Gal4/UAS-AstC (12-14)	ns	0.38
	72 HAE		
	AstC1/+,AstC-Gal4/+ (12-14) vs. AstC1/AstC1 (12-14)	ns	0.21
	AstC1/+,AstC-Gal4/+ (12-14) vs. AstC1/AstC1>AstC/AstC (12-14)	ns	0.24
	AstC1/AstC1 (12-14) vs. AstC1/AstC1,AstC-Gal4/UAS-AstC (12-14)	***	<0.001
	96 HAE		
	AstC1/+,AstC-Gal4/+ (12-14) vs. AstC1/AstC1 (12-14)	**	0.006
	AstC1/+,AstC-Gal4/+ (12-14) vs. AstC1/AstC1>AstC/AstC (12-14)	*	0.02
	AstC1/AstC1 (12-14) vs. AstC1/AstC1,AstC-Gal4/UAS-AstC (12-14)	***	<0.001
	120 HAE		
	AstC1/+,AstC-Gal4/+ (12-14) vs. AstC1/AstC1 (12-14)	ns	0.95
	AstC1/+,AstC-Gal4/+ (12-14) vs. AstC1/AstC1>AstC/AstC (12-14)	ns	0.83
	AstC1/AstC1 (12-14) vs. AstC1/AstC1,AstC-Gal4/UAS-AstC (12-14)	ns	0.06

Fig. 3f	One-way ANOVA followed by Tukey's multiple comparison test		
		Summary	Adjusted P Value
	JHAMT-Gal4>UAS-AstCR1 vs. UAS-AstCR1>	ns	0.99
	JHAMT-Gal4>UAS-AstCR1 vs. JHAMT-Gal4>UAS-AstCR2	ns	0.62
	JHAMT-Gal4>UAS-AstCR1 vs. UAS-AstCR2>	ns	0.99
	JHAMT-Gal4>UAS-AstCR1 vs. JHAMT-Gal4>UAS-AstCR1R2	**	0.002
	JHAMT-Gal4>UAS-AstCR1 vs. UAS-AstCR1R2>	ns	>0.99

	JHAMT>R1-IR (8-11) vs. R1-IR> (8-11)	***	<0.001
	JHAMT>R1-IR (8-11) vs. JHAMT>R2-IR (8-11)	ns	>0.99
	JHAMT>R1-IR (8-11) vs. R2-IR> (8-11)	**	0.001
	JHAMT>R1-IR (8-11) vs. JHAMT> (8-11)	***	<0.001
	R1-IR> (8-11) vs. JHAMT>R2-IR (8-11)	***	<0.001
	R1-IR> (8-11) vs. R2-IR> (8-11)	ns	>0.99
	R1-IR> (8-11) vs. JHAMT> (8-11)	ns	>0.99
	JHAMT>R2-IR (8-11) vs. R2-IR> (8-11)	***	<0.001
	JHAMT>R2-IR (8-11) vs. JHAMT> (8-11)	***	<0.001
	R2-IR> (8-11) vs. JHAMT> (8-11)	ns	>0.99
	24 HAE		
	JHAMT>R1-IR (8-11) vs. R1-IR> (8-11)	***	<0.001
	JHAMT>R1-IR (8-11) vs. JHAMT>R2-IR (8-11)	ns	>0.99
	JHAMT>R1-IR (8-11) vs. R2-IR> (8-11)	***	<0.001
	JHAMT>R1-IR (8-11) vs. JHAMT> (8-11)	***	<0.001
	R1-IR> (8-11) vs. JHAMT>R2-IR (8-11)	*	0.04
	R1-IR> (8-11) vs. R2-IR> (8-11)	ns	>0.99
	R1-IR> (8-11) vs. JHAMT> (8-11)	*	0.03
	JHAMT>R2-IR (8-11) vs. R2-IR> (8-11)	**	0.004
	JHAMT>R2-IR (8-11) vs. JHAMT> (8-11)	***	<0.001
	R2-IR> (8-11) vs. JHAMT> (8-11)	ns	0.24
	12 HAE		
	JHAMT>R1-IR (12-14) vs. R1-IR> (12-14)	ns	>0.99
	JHAMT>R1-IR (12-14)vs. JHAMT>R2-IR (12-14)	ns	>0.99
	JHAMT>R1-IR (12-14) vs. R2-IR> (12-14)	ns	>0.99
	JHAMT>R1-IR (12-14) vs. JHAMT> (12-14)	ns	>0.99
	R1-IR> (12-14)vs. JHAMT>R2-IR (12-14)	ns	>0.99
	R1-IR> (12-14) vs. R2-IR> (12-14)	ns	>0.99
	R1-IR> (12-14) vs. JHAMT> (12-14)	ns	>0.99
	JHAMT>R2-IR (12-14)vs. R2-IR> (12-14)	ns	>0.99
	JHAMT>R2-IR (12-14)vs. JHAMT> (12-14)	ns	>0.99
	R2-IR> (12-14) vs. JHAMT> (12-14)	ns	>0.99
	24 HAE		
	JHAMT>R1-IR (12-14) vs. R1-IR> (12-14)	ns	>0.99
	JHAMT>R1-IR (12-14)vs. JHAMT>R2-IR (12-14)	ns	>0.99
	JHAMT>R1-IR (12-14) vs. R2-IR> (12-14)	ns	>0.99
	JHAMT>R1-IR (12-14) vs. JHAMT> (12-14)	*	0.04
	R1-IR> (12-14)vs. JHAMT>R2-IR (12-14)	ns	>0.99
	R1-IR> (12-14) vs. R2-IR> (12-14)	ns	>0.99
	R1-IR> (12-14) vs. JHAMT> (12-14)	ns	>0.99
	JHAMT>R2-IR (12-14)vs. R2-IR> (12-14)	ns	>0.99
	JHAMT>R2-IR (12-14)vs. JHAMT> (12-14)	ns	0.29
	R2-IR> (12-14) vs. JHAMT> (12-14)	ns	0.17

	Two-way ANOVA followed by Bonferroni post tests		
	12 -24 HAE	Summary	Adjusted P Value
	JHAMT>R1-IR (8-11)	***	<0.001
	R1-IR> (8-11)	***	<0.001
	JHAMT>R2-IR (8-11)	ns	0.13
	R2-IR> (8-11)	*	0.02

	JHAMT-Gal4>UAS-AstCR1 vs. JHAMT-Gal4>	ns	>0.99
	UAS-AstCR1> vs. JHAMT-Gal4>UAS-AstCR2	*	0.05
	UAS-AstCR1> vs. UAS-AstCR2>	ns	0.47
	UAS-AstCR1> vs. JHAMT-Gal4>UAS-AstCR1R2	***	<0.001
	UAS-AstCR1> vs. UAS-AstCR1R2>	ns	0.99
	UAS-AstCR1> vs. JHAMT-Gal4>	ns	0.56
	JHAMT-Gal4>UAS-AstCR2 vs. UAS-AstCR2>	ns	0.86
	JHAMT-Gal4>UAS-AstCR2 vs. JHAMT-Gal4>UAS-AstCR1R2	*	0.02
	JHAMT-Gal4>UAS-AstCR2 vs. UAS-AstCR1R2>	ns	0.08
	JHAMT-Gal4>UAS-AstCR2 vs. JHAMT-Gal4>	ns	0.89
	UAS-AstCR2> vs. JHAMT-Gal4>UAS-AstCR1R2	***	<0.001
	UAS-AstCR2> vs. UAS-AstCR1R2>	ns	0.79
	UAS-AstCR2> vs. JHAMT-Gal4>	ns	>0.99
	JHAMT-Gal4>UAS-AstCR1R2 vs. UAS-AstCR1R2>	***	<0.001
	JHAMT-Gal4>UAS-AstCR1R2 vs. JHAMT-Gal4>	***	<0.001
	UAS-AstCR1R2> vs. JHAMT-Gal4>	ns	0.86

Fig. 4a	One-way ANOVA followed by Tukey's multiple comparison test		
		Summary	Adjusted P Value
	mTh/Tric virgin vs. mTh/Tric mated	**	0.006
	mTh/Tric virgin vs. mTh/Tric mated SP0/+	*	0.03
	mTh/Tric virgin vs. mTh/Tric mated SP0/Δ^{130}	ns	0.98
	mTh/Tric mated vs. mTh/Tric mated SP0/+	ns	>0.99
	mTh/Tric mated vs. mTh/Tric mated SP0/Δ^{130}	**	0.006
	mTh/Tric mated SP0/+ vs. mTh Tric mated SP0/Δ^{130}	*	0.02

Fig. 4b	One-way ANOVA followed by Tukey's multiple comparison test		
	Stage 8	Summary	Adjusted P Value
	dTrpA1> vs. AstC-D>dTrpA1	ns	>0.99
	dTrpA1> vs. AstC-D>	ns	0.64
	AstC-D>dTrpA1 vs. AstC-D>	ns	0.64
	Stage 9		
	dTrpA1> vs. AstC-D>dTrpA1	ns	0.12
	dTrpA1> vs. AstC-D>	ns	0.28
	AstC-D>dTrpA1 vs. AstC-D>	ns	0.86
	Stage 10		
	dTrpA1> vs. AstC-D>dTrpA1	***	<0.001
	dTrpA1> vs. AstC-D>	ns	0.47
	AstC-D>dTrpA1 vs. AstC-D>	***	<0.001
	Stage 11		
	dTrpA1> vs. AstC-D>dTrpA1	ns	0.12
	dTrpA1> vs. AstC-D>	ns	>0.99
	AstC-D>dTrpA1 vs. AstC-D>	ns	0.12
	Stage 12		
	dTrpA1> vs. AstC-D>dTrpA1	ns	0.46
	dTrpA1> vs. AstC-D>	ns	0.31
	AstC-D>dTrpA1 vs. AstC-D>	*	0.04
	Stage 13		
	dTrpA1> vs. AstC-D>dTrpA1	ns	>0.99
	dTrpA1> vs. AstC-D>	ns	0.11
	AstC-D>dTrpA1 vs. AstC-D>	ns	0.11
	Stage 14		
	dTrpA1> vs. AstC-D>dTrpA1	ns	0.84
	dTrpA1> vs. AstC-D>	ns	0.07
	AstC-D>dTrpA1 vs. AstC-D>	*	0.03
	ovi		
	dTrpA1> vs. AstC-D>dTrpA1	ns	0.26
	dTrpA1> vs. AstC-D>	ns	0.6
	AstC-D>dTrpA1 vs. AstC-D>	*	0.05

Fig. 4c	One-way ANOVA followed by Tukey's multiple comparison test		
	Stage 8	Summary	Adjusted P Value
	Jhamt>ETHR IR vs. Jhamt>	ns	0.68
	Jhamt>ETHR IR vs. ETHR IR>	ns	0.63

	JHAMT> (8-11)	ns	>0.99
	12 -24 HAE		
	JHAMT>R1-IR (12-14)	***	<0.001
	R1-IR> (12-14)	***	<0.001
	JHAMT>R2-IR (12-14)	***	<0.001
	R2-IR> (12-14)	***	<0.001
	JHAMT> (12-14)	***	<0.001

Fig. 5b	One-way ANOVA followed by Tukey's multiple comparison test		
		Summary	Adjusted P Value
	SAG VT>TrpA1 vs. SAG VT>	***	<0.001
	SAG VT>TrpA1 vs. Trpa1>	***	<0.001
	SAG VT>TrpA1 vs. Met SAG VT>TrpA1	***	<0.001
	SAG VT>TrpA1 vs. AstC1/+, SAG>Trpa1	ns	0.39
	SAG VT>TrpA1 vs. AstC1/AstC1, SAG>Trpa1	***	<0.001
	SAG VT> vs. Trpa1>	ns	0.8
	SAG VT> vs. Met SAG VT>TrpA1	ns	0.98
	SAG VT> vs. AstC1/+, SAG>Trpa1	***	<0.001
	SAG VT> vs. AstC1/AstC1, SAG>Trpa1	ns	0.64
	Trpa1> vs. Met SAG VT>TrpA1	ns	>0.99
	Trpa1> vs. AstC1/+, SAG>Trpa1	***	<0.001
	Trpa1> vs. AstC1/AstC1, SAG>Trpa1	ns	0.1
	Met SAG VT>TrpA1 vs. AstC1/+, SAG>Trpa1	***	<0.001
	Met SAG VT>TrpA1 vs. AstC1/AstC, SAG>Trpa1	ns	0.29
	AstC1/+, SAG>Trpa1 vs. AstC1/AstC1, SAG>Trpa1	***	<0.001

Fig. 5c	One-way ANOVA followed by Tukey's multiple comparison test		
		Summary	Adjusted P Value
	50405-LexA>LexAop-Trpa1 vs. LexAop-Trpa1/UAS-Kir2.1>	***	<0.001
	50405-LexA>LexAop-Trpa1 vs. AstC-D-Gal4>UAS-Kir2.1	***	<0.001
	50405-LexA>LexAop-Trpa1 vs. 50405:AstC-D>Kir2.1:Trpa1	*	0.02
	50405-LexA>LexAop-Trpa1 vs. 50405-LexA>	**	0.001
	50405-LexA>LexAop-Trpa1 vs. AstC-D-Gal4>	**	0.009
	LexAop-Trpa1/UAS-Kir2.1> vs. AstC-D-Gal4>UAS-Kir2.1	ns	0.99
	LexAop-Trpa1/UAS-Kir2.1> vs. 50405:AstC-D>Kir2.1:Trpa1	ns	0.25
	LexAop-Trpa1/UAS-Kir2.1> vs. 50405-LexA>+	ns	0.48
	LexAop-Trpa1/UAS-Kir2.1> vs. AstC-D-Gal4>+	ns	0.41
	AstC-D-Gal4>UAS-Kir2.1 vs. 50405:AstC-D>Kir2.1:Trpa1	ns	0.65
	AstC-D-Gal4>UAS-Kir2.1 vs. 50405-LexA>	ns	0.89
	AstC-D-Gal4>UAS-Kir2.1 vs. AstC-D-Gal4>	ns	0.82
	50405:AstC-D>Kir2.1:Trpa1 vs. 50405-LexA>	ns	>0.99
	50405:AstC-D>Kir2.1:Trpa1 vs. AstC-D-Gal4>	ns	>0.99
	50405-LexA> vs. AstC-D-Gal4>	ns	>0.99

Fig. 5d	Two-way ANOVA followed by Bonferroni post tests		
	12 HAE	Summary	Adjusted P Value
	SAG1>Kir2.1 (8-11) vs. SAG1> (8-11)	**	0.005
	SAG1>Kir2.1 (8-11) vs. D>trpa1 (8-11)	***	<0.001
	SAG1>Kir2.1 (8-11) vs. D> (8-11)	*	0.02
	SAG1>Kir2.1 (8-11) vs. SAG1>Kir2.1/D>trpa1(8-11)	***	<0.001
	SAG1>Kir2.1 (8-11) vs. trpa1/Kir2.1> (8-11)	*	0.04
	SAG1> (8-11) vs. D>trpa1 (8-11)	ns	>0.99
	SAG1> (8-11) vs. D> (8-11)	ns	>0.99
	SAG1> (8-11) vs. SAG1>Kir2.1/D>trpa1(8-11)	ns	>0.99
	SAG1> (8-11) vs. trpa1/Kir2.1> (8-11)	ns	>0.99
	D>trpa1 (8-11) vs. D> (8-11)	ns	>0.99
	D>trpa1 (8-11) vs. SAG1>Kir2.1/D>trpa1(8-11)	ns	>0.99
	D>trpa1 (8-11) vs. trpa1/Kir2.1> (8-11)	ns	>0.99
	D> (8-11) vs. SAG1>Kir2.1/D>trpa1(8-11)	ns	>0.99
	D> (8-11) vs. trpa1/Kir2.1> (8-11)	ns	>0.99
	SAG1>Kir2.1/D>trpa1(8-11) vs. trpa1/Kir2.1> (8-11)	ns	>0.99
	24 HAE		
	SAG1>Kir2.1 (8-11) vs. SAG1> (8-11)	***	<0.001

	Jhamt> vs. ETHR IR>	ns	>0.99
	Stage 9		
	Jhamt>ETHR IR vs. Jhamt>	ns	0.73
	Jhamt>ETHR IR vs. ETHR IR>	ns	0.94
	Jhamt> vs. ETHR IR>	ns	0.52
	Stage 10		
	Jhamt>ETHR IR vs. Jhamt>	**	0.001
	Jhamt>ETHR IR vs. ETHR IR>	**	0.009
	Jhamt> vs. ETHR IR>	ns	0.83
	Stage 11		
	Jhamt>ETHR IR vs. Jhamt>	ns	0.94
	Jhamt>ETHR IR vs. ETHR IR>	ns	0.75
	Jhamt> vs. ETHR IR>	ns	0.54
	Stage 12		
	Jhamt>ETHR IR vs. Jhamt>	ns	0.51
	Jhamt>ETHR IR vs. ETHR IR>	ns	0.67
	Jhamt> vs. ETHR IR>	ns	0.97
	Stage 13		
	Jhamt>ETHR IR vs. Jhamt>	ns	0.89
	Jhamt>ETHR IR vs. ETHR IR>	ns	0.9
	Jhamt> vs. ETHR IR>	ns	0.64
	Stage 14		
	Jhamt>ETHR IR vs. Jhamt>	ns	0.25
	Jhamt>ETHR IR vs. ETHR IR>	ns	0.17
	Jhamt> vs. ETHR IR>	ns	0.97
	ovi		
	Jhamt>ETHR IR vs. Jhamt>	**	0.009
	Jhamt>ETHR IR vs. ETHR IR>	ns	0.05
	Jhamt> vs. ETHR IR>	ns	0.8

Fig. 4d	One-way ANOVA followed by Tukey's multiple comparison test		
	Stage 8	Summary	Adjusted P Value
	SAG1>dTrpA1 vs. SAG1>	ns	0.6
	SAG1>dTrpA1 vs.dTrpA1>	ns	0.92
	SAG1> vs. dTrpA1>	ns	0.83
	Stage 9		
	SAG1>dTrpA1 vs. SAG1>	ns	>0.99
	SAG1>dTrpA1 vs.dTrpA1>	ns	0.76
	SAG1> vs. dTrpA1>	ns	0.76
	Stage 10		
	SAG1>dTrpA1 vs. SAG1>	***	<0.001
	SAG1>dTrpA1 vs.dTrpA1>	***	<0.001
	SAG1> vs. dTrpA1>	ns	0.57
	Stage 11		
	SAG1>dTrpA1 vs. SAG1>	ns	0.96
	SAG1>dTrpA1 vs.dTrpA1>	ns	0.51
	SAG1> vs. dTrpA1>	ns	0.36
	Stage 12		
	SAG1>dTrpA1 vs. SAG1>	ns	0.95
	SAG1>dTrpA1 vs.dTrpA1>	ns	0.61
	SAG1> vs. dTrpA1>	ns	0.8
	Stage 13		
	SAG1>dTrpA1 vs. SAG1>	ns	0.59
	SAG1>dTrpA1 vs.dTrpA1>	ns	0.41
	SAG1> vs. dTrpA1>	ns	0.94
	Stage 14		
	SAG1>dTrpA1 vs. SAG1>	***	<0.001
	SAG1>dTrpA1 vs.dTrpA1>	***	<0.001
	SAG1> vs. dTrpA1>	ns	0.55
	ovi		
	SAG1>dTrpA1 vs. SAG1>	***	<0.001
	SAG1>dTrpA1 vs.dTrpA1>	***	<0.001
	SAG1> vs. dTrpA1>	ns	0.77

Fig. 5a	Two-way ANOVA followed by Bonferroni post tests		
	12 HAE	Summary	Adjusted P Value
	SAG>Kir2.1 (8-11) vs. *SAG-Gal4>* (8-11)	*	0.02
	SAG>Kir2.1 (8-11) vs. *UAS-Kir2.1>* (8-11)	***	<0.001
	SAG-Gal4> (8-11) vs. *UAS-Kir2.1>* (8-11)	ns	0.96
	24 hours	Summary	Adjusted P Value

	SAG1>Kir2.1 (8-11) vs. *D>trpa1* (8-11)	***	<0.001
	SAG1>Kir2.1 (8-11) vs. *D>* (8-11)	***	<0.001
	SAG1>Kir2.1 (8-11) vs. *SAG1>Kir2.1/D>trpa1*(8-11)	***	<0.001
	SAG1>Kir2.1 (8-11) vs. *trpa1/Kir2.1>* (8-11)	**	0.006
	SAG1> (8-11) vs. *D>trpa1* (8-11)	*	0.02
	SAG1> (8-11) vs. *D>* (8-11)	ns	>0.99
	SAG1> (8-11) vs. *SAG1>Kir2.1/D>trpa1*(8-11)	ns	0.85
	SAG1> (8-11) vs. *trpa1/Kir2.1>* (8-11)	ns	>0.99
	D>trpa1 (8-11) vs. *D>* (8-11)	ns	0.17
	D>trpa1 (8-11) vs. *SAG1>Kir2.1/D>trpa1*(8-11)	ns	>0.99
	D>trpa1 (8-11) vs. *trpa1/Kir2.1>* (8-11)	*	0.01
	D> (8-11) vs. *SAG1>Kir2.1/D>trpa1*(8-11)	ns	>0.99
	D> (8-11) vs. *trpa1/Kir2.1>* (8-11)	ns	>0.99
	SAG1>Kir2.1/D>trpa1(8-11) vs. *trpa1/Kir2.1>* (8-11)	ns	0.58
	12 HAE		
	SAG1>Kir2.1 (12-14) vs. *SAG1>* (12-14)	ns	>0.99
	SAG1>Kir2.1 (12-14) vs. *D>trpa1* (12-14)	ns	>0.99
	SAG1>Kir2.1 (12-14) vs. *D>* (12-14)	ns	>0.99
	SAG1>Kir2.1 (12-14) vs. *SAG1>Kir2.1/D>trpa1*(12-14)	ns	>0.99
	SAG1>Kir2.1 (12-14) vs. *trpa1/Kir2.1>* (12-14)	ns	>0.99
	SAG1> (12-14) vs. *D>trpa1* (12-14)	ns	>0.99
	SAG1> (12-14) vs. *D>* (12-14)	ns	>0.99
	SAG1> (12-14) vs. *SAG1>Kir2.1/D>trpa1*(12-14)	ns	>0.99
	SAG1> (12-14) vs. *trpa1/Kir2.1>* (12-14)	ns	>0.99
	D>trpa1 (12-14) vs. *D>* (12-14)	ns	>0.99
	D>trpa1 (12-14) vs. *SAG1>Kir2.1/D>trpa1*(12-14)	ns	>0.99
	D>trpa1 (12-14) vs. *trpa1/Kir2.1>* (12-14)	ns	>0.99
	D> (12-14) vs. *SAG1>Kir2.1/D>trpa1*(12-14)	ns	>0.99
	D> (12-14) vs. *trpa1/Kir2.1>* (12-14)	ns	>0.99
	SAG1>Kir2.1/D>trpa1(12-14) vs. *trpa1/Kir2.1>* (12-14)	ns	>0.99
	24 HAE		
	SAG1>Kir2.1 (12-14) vs. *SAG1>* (12-14)	ns	>0.99
	SAG1>Kir2.1 (12-14) vs. *D>trpa1* (12-14)	ns	0.53
	SAG1>Kir2.1 (12-14) vs. *D>* (12-14)	ns	>0.99
	SAG1>Kir2.1 (12-14) vs. *SAG1>Kir2.1/D>trpa1*(12-14)	ns	>0.99
	SAG1>Kir2.1 (12-14) vs. *trpa1/Kir2.1>* (12-14)	ns	0.3
	SAG1> (12-14) vs. *D>trpa1* (12-14)	ns	0.19
	SAG1> (12-14) vs. *D>* (12-14)	ns	>0.99
	SAG1> (12-14) vs. *SAG1>Kir2.1/D>trpa1*(12-14)	ns	>0.99
	SAG1> (12-14) vs. *trpa1/Kir2.1>* (12-14)	ns	0.71
	D>trpa1 (12-14) vs. *D>* (12-14)	*	0.02
	D>trpa1 (12-14) vs. *SAG1>Kir2.1/D>trpa1*(12-14)	ns	>0.99
	D>trpa1 (12-14) vs. *trpa1/Kir2.1>* (12-14)	***	<0.001
	D> (12-14) vs. *SAG1>Kir2.1/D>trpa1*(12-14)	ns	>0.99
	D> (12-14) vs. *trpa1/Kir2.1>* (12-14)	ns	>0.99
	SAG1>Kir2.1/D>trpa1(12-14) vs. *trpa1/Kir2.1>* (12-14)	ns	0.09
	Two-way ANOVA followed by Bonferroni post tests		
	12 - 24 HAE	Summary	Adjusted P Value
	SAG1>Kir2.1 (8-11)	***	<0.001
	SAG1> (8-11)	***	<0.001
	D>trpa1 (8-11)	*	0.03
	D> (8-11)	**	0.003
	SAG1>Kir2.1/D>trpa1(8-11)	**	0.005
	trpa1/Kir2.1> (8-11)	***	<0.001
	12 -24 HAE		
	SAG1>Kir2.1 (12-14)	***	<0.001
	SAG1> (12-14)	***	<0.001
	D>trpa1 (12-14)	**	0.001
	D> (12-14)	***	<0.001
	SAG1>Kir2.1/D>trpa1(12-14)	***	<0.001
	trpa1/Kir2.1> (12-14)	***	<0.001

Fig. 5e	One-way ANOVA followed by Tukey's multiple comparison test		
		Summary	Adjusted P Value
	SAG1/Tric 0D vs. SAG1/Tric 1D	ns	0.77
	SAG1/Tric 0D vs. SAG1/Tric 2D	*	0.02
	SAG1/Tric 0D vs. SAG1/Tric 3D	***	<0.001
	SAG1/Tric 1D vs. SAG1/Tric 2D	ns	0.15

	SAG>Kir2.1 (8-11) vs. SAG-Gal4> (8-11)	*	0.03
	SAG>Kir2.1 (8-11) vs. UAS-Kir2.1> (8-11)	***	<0.001
	SAG-Gal4> (8-11) vs. UAS-Kir2.1> (8-11)	ns	0.67
	12 HAE	Summary	Adjusted P Value
	SAG>Kir2.1 (12-14) vs. SAG-Gal4> (12-14)	ns	>0.99
	SAG>Kir2.1 (12-14) vs. UAS-Kir2.1> (12-14)	ns	>0.99
	SAG-Gal4> (12-14) vs. UAS-Kir2.1> (12-14)	ns	>0.99
	24 hours	Summary	Adjusted P Value
	SAG>Kir2.1 (12-14) vs. SAG-Gal4> (12-14)	ns	0.12
	SAG>Kir2.1 (12-14) vs. UAS-Kir2.1> (12-14)	ns	0.79
	SAG-Gal4> (12-14) vs. UAS-Kir2.1> (12-14)	ns	0.69
	Two-way ANOVA followed by Bonferroni post tests		
	12 - 24 HAE	Summary	Adjusted P Value
	SAG>Kir2.1 (8-11)	***	<0.001
	SAG-Gal4> (8-11)	***	<0.001
	UAS-Kir2.1> (8-11)	***	<0.001
	12 -24 HAE	Summary	Adjusted P Value
	SAG>Kir2.1 (12-14)	***	<0.001
	SAG-Gal4> (12-14)	***	<0.001
	UAS-Kir2.1> (12-14)	***	<0.001

Supplementary Fig.1g	Two-way ANOVA followed by Bonferroni post tests		
	12 HAE	Summary	Adjusted P Value
	AstC-D>Shi ts vs. AstC-D> (8-11)	**	0.002
	AstC-D>Shi ts vs. Kir2.1> (8-11)	**	0.006
	AstC-D>vs. Shi ts> 8-11)	ns	>0.99
	24 HAE		
	AstC-D>Shi ts vs. AstC-D> (8-11)	***	<0.001
	AstC-D>Shi ts vs. Kir2.1> (8-11)	***	<0.001
	AstC-D>vs. Shi ts> (8-11)	*	0.04
	12 HAE		
	AstC-D>Shi ts vs. AstC-D> (12-14)	ns	>0.99
	AstC-D>Shi ts vs. Kir2.1> (12-14)	ns	>0.99
	AstC-D>vs. Shi ts> (12-14)	ns	>0.99
	24 HAE		
	AstC-D>Shi ts vs. AstC-D> (12-14)	*	0.05
	AstC-D>Shi ts vs. Kir2.1> (12-14)	***	<0.001
	AstC-D>vs. Shi ts> (12-14)	ns	0.43
	Two-way ANOVA followed by Bonferroni post tests		
	12 -24 HAE		
	AstC-D>Shi (8-11)	***	<0.001
	AstC-D>(8-11)	***	<0.001
	Shit ts>(8-11)	***	<0.001
	12 -24 HAE		
	AstC-D>Shi (12-14)	***	<0.001
	AstC-D>(12-14)	***	<0.001
	Shit ts>(12-14)	***	<0.001

Supplementary Fig.4c	One-way ANOVA followed by Tukey's multiple comparison test		
	Tasting events	Summary	Adjusted P Value
	AstC-D>Kir2.1 vs. AstC-D>	ns	0.67
	AstC-D>Kir2.1 vs. Kir2.1>	ns	0.62
	AstC-D>vs. Kir2.1>	ns	>0.99
	Interval		
	AstC-D>Kir2.1 vs. AstC-D>	ns	0.7
	AstC-D>Kir2.1 vs. Kir2.1>	ns	0.91
	AstC-D>vs. Kir2.1>	ns	0.92
	Mean duration		
	AstC-D>Kir2.1 vs. AstC-D>	ns	0.35
	AstC-D>Kir2.1 vs. Kir2.1>	ns	0.33
	AstC-D>vs. Kir2.1>	ns	>0.99

Supplementary Fig.8a	One-way ANOVA followed by Tukey's multiple comparison test		
	Stage 8	Summary	Adjusted P Value
	dTrpa1> vs. AstC-D>dTrpa1	ns	0.08
	dTrpa1> vs. AstC-D>	ns	0.31
	AstC-D>vs. AstC-D>dTrpa1	ns	0.7
	Stage 9		
	dTrpa1> vs. AstC-D>dTrpa1	ns	0.94
	dTrpa1> vs. AstC-D>	ns	0.13
	AstC-D>vs. AstC-D>dTrpa1	ns	0.14
	Stage 10		
	dTrpa1> vs. AstC-D>dTrpa1	***	<0.001
	dTrpa1> vs. AstC-D>	ns	0.65
	AstC-D>vs. AstC-D>dTrpa1	***	<0.001
	Stage 11		
	dTrpa1> vs. AstC-D>dTrpa1	*	0.02
	dTrpa1> vs. AstC-D>	***	<0.001
	AstC-D>vs. AstC-D>dTrpa1	**	0.005
	Stage 12		

	SAG1/Tric 1D vs. SAG1/Tric 3D	**	0.004
	SAG1/Tric 2D vs. SAG1/Tric 3D	ns	0.55

Supplementary Fig.1d	One-way ANOVA followed by Tukey's multiple comparison test		
		Summary	Adjusted P Value
	AstC01/+>CNMa/+ (8-11) vs. AstC01/AstC01>CNMa/+(8-11)	*	0.02
	AstC01/+>CNMa/+ (8-11) vs. AstC01/AstC01>CNMa/AstC(8-11)	**	0.003
	AstC01/AstC01>CNMa/+(8-11) vs. AstC01/AstC01>CNMa/AstC(8-11)	ns	0.68
	AstC01/+>CNMa/+ (12-14) vs. AstC01/AstC01>CNMa/+(12-14)	ns	0.94
	AstC01/+>CNMa/+ (12-14) vs. AstC01/AstC01>CNMa/AstC(12-14)	ns	0.51
	AstC01/AstC01>CNMa/+(12-14) vs. AstC01/AstC01>CNMa/AstC(12-14)	ns	0.32

Supplementary Fig.1e	One-way ANOVA followed by Tukey's multiple comparison test		
		Summary	Adjusted P Value
	AstC-D>dTrpa1 vs. AstC-D>	ns	0.76
	AstC-D>drpa1 vs. dTrpa1>	ns	0.68
	AstC-D> vs. dTpra1>	ns	0.3

Supplementary Fig.1f	One-way ANOVA followed by Tukey's multiple comparison test		
		Summary	Adjusted P Value
	AstC-D>Kir2.1 vs. AstC-D>	ns	0.9
	AstC-D>Kir2.1 vs. Kir2.1>	ns	0.95
	AstC-D>vs. Kir2.1>	ns	0.75

Supplementary Fig.2h	One-way ANOVA followed by Tukey's multiple comparison test		
		Summary	Adjusted P Value
	mTh/Tric 0D vs. mTh/Tric 1D	ns	>0.99
	mTh/Tric 0D vs. mTh/Tric 2D	ns	>0.99
	mTh/Tric 0D vs. mTh/Tric 3D	ns	0.84
	mTh/Tric 0D vs. mTh/Tric -1D	ns	>0.99
	mTh/Tric 1D vs. mTh/Tric 2D	ns	>0.99
	mTh/Tric 1D vs. mTh/Tric 3D	ns	0.94
	mTh/Tric 1D vs. mTh/Tric -1D	ns	>0.99
	mTh/Tric 2D vs. mTh/Tric 3D	ns	0.88
	mTh/Tric 2D vs. mTh/Tric -1D	ns	>0.99
	mTh/Tric 3D vs. mTh/Tric -1D	ns	0.78

Supplementary Fig.3a	One-way ANOVA followed by Tukey's multiple comparison test		
		Summary	Adjusted P Value
	pupa vs. W	**	0.005
	pupa vs. AstC-D>Trpa1	ns	0.2
	W vs. AstC-D>Trpa1	*	0.02
Supplementary Fig.3b	One-way ANOVA followed by Tukey's multiple comparison test		
		Summary	Adjusted P Value
	w1118 pupa vs. w1118 D1 adult	***	<0.001
	w1118 pupa vs. Trpa1> D1 adult	***	<0.001
	w1118 pupa vs. AstC-D> D1 adult	***	<0.001
	w1118 pupa vs. AstC-D>Trpa1	***	<0.001
	w1118 D1 adult vs. Trpa1> D1 adult	ns	0.14
	w1118 D1 adult vs. AstC-D> D1 adult	ns	0.29
	w1118 D1 adult vs. AstC-D>Trpa1	ns	0.09
	Trpa1> D1 adult vs. AstC-D> D1 adult	ns	0.98
	Trpa1> D1 adult vs. AstC-D>Trpa1	***	<0.001
	AstC-D> D1 adult vs. AstC-D>Trpa1	***	<0.001
Supplementary Fig.6a	One-way ANOVA followed by Tukey's multiple comparison test		
	Dilp2 intensity	Summary	Adjusted P Value
	dTrpa1> vs. AstC-D>	ns	0.05
	dTrpa1> vs. AstC-D>dTrpa1	**	0.007
	AstC-D>vs. AstC-D>dTrpa1	ns	0.83
Supplementary Fig.6b	One-way ANOVA followed by Tukey's multiple comparison test		
		Summary	Adjusted P Value
	AstC-D>Nachbac vs. AstC-D>	ns	0.86
	AstC-D>Nachbac vs. Nachbac>	ns	0.08
	AstC-D> vs. Nachbac>	*	0.03
Supplementary Fig.8b	One-way ANOVA followed by Tukey's multiple comparison test		
	Stage 8	Summary	Adjusted P Value
	Shi ts> vs. AstC-D>Shi ts	ns	0.92
	Shi ts> vs. AstC-D>	ns	0.14
	AstC-D>vs. AstC-D>Shi ts	ns	0.06
	Stage 9		
	Shi ts> vs. AstC-D>Shi ts	ns	0.11
	Shi ts> vs. AstC-D>	ns	0.4
	AstC-D>vs. AstC-D>Shi ts	**	0.007
	Stage 10		
	Shi ts> vs. AstC-D>Shi ts	ns	0.7
	Shi ts> vs. AstC-D>	*	0.05
	AstC-D>vs. AstC-D>Shi ts	**	0.008
	Stage 11		
	Shi ts> vs. AstC-D>Shi ts	ns	0.89
	Shi ts> vs. AstC-D>	ns	0.08
	AstC-D>vs. AstC-D>Shi ts	ns	0.18
	Stage 12		

	dTrpa1> vs. AstC-D>dTrpa1	ns	0.06
	dTrpa1> vs. AstC-D>	ns	0.28
	AstC-D>vs. AstC-D>dTrpa1	***	<0.001
	Stage 13		
	dTrpa1> vs. AstC-D>dTrpa1	ns	0.85
	dTrpa1> vs. AstC-D>	ns	0.87
	AstC-D>vs. AstC-D>dTrpa1	ns	>0.99
	Stage 14		
	dTrpa1> vs. AstC-D>dTrpa1	***	<0.001
	dTrpa1> vs. AstC-D>	ns	0.51
	AstC-D>vs. AstC-D>dTrpa1	***	<0.001
	ovi		
	dTrpa1> vs. AstC-D>dTrpa1	**	0.003
	dTrpa1> vs. AstC-D>	ns	>0.99
	AstC-D>vs. AstC-D>dTrpa1	**	0.001

	Shi ts> vs. AstC-D>Shi ts	ns	0.68
	Shi ts> vs. AstC-D>	ns	0.91
	AstC-D>vs. AstC-D>Shi ts	ns	0.42
	Stage 13		
	Shi ts> vs. AstC-D>Shi ts	ns	0.81
	Shi ts> vs. AstC-D>	ns	0.69
	AstC-D>vs. AstC-D>Shi ts	ns	0.33
	Stage 14		
	Shi ts> vs. AstC-D>Shi ts	ns	0.87
	Shi ts> vs. AstC-D>	ns	0.23
	AstC-D>vs. AstC-D>Shi ts	ns	0.09
	ovi		
	Shi ts> vs. AstC-D>Shi ts	ns	0.25
	Shi ts> vs. AstC-D>	ns	0.49
	AstC-D>vs. AstC-D>Shi ts	*	0.03

Supplementary Table 2. A list of detailed genotypes, age and sex of flies examined in this study.

Figure	Full genotypes	Age and Sex
Fig. 1a	w^{1118}(I); *AstC-Gal4, AstC*^{1}> +(II)	0~120 HAE; virgin females
	w^{1118}(I); *AstC*^{1}>*AstC*^{1}(II)	0~120 HAE; virgin females
	w^{1118}(I); *AstC-Gal4, AstC*^{1},*AstC*^{1}(II) > *UAS-AstC* (III)	0~120 HAE; virgin females
Fig. 1c	w^{1118}(I); *AstC-A-Gal4>UAS-dTrpA1*(II)	3 days old; virgin females
	w^{1118}(I); *AstC-A-Gal4>+*(II)	3 days old; virgin females
	w^{1118}(I); *AstC-B-Gal4>UAS-dTrpA1*(II)	3 days old; virgin females
	w^{1118}(I); *AstC-B-Gal4>+*(II)	3 days old; virgin females
	w^{1118}(I); *AstC-C-Gal4>UAS-dTrpA1*(II)	3 days old; virgin females
	w^{1118}(I); *AstC-C-Gal4>+*(II)	3 days old; virgin females
	w^{1118}(I); *AstC-D-Gal4>UAS-dTrpA1*(II)	3 days old; virgin females
	w^{1118}(I); *AstC-D-Gal4>+*(II)	3 days old; virgin females
	w^{1118}(I); *AstC-E-Gal4>UAS-dTrpA1*(II)	3 days old; virgin females
	w^{1118}(I); *AstC-E-Gal4>+*(II)	3 days old; virgin females
	w^{1118}(I);*UAS-dTrpA1>+*(II)	3 days old; virgin females
Fig. 1d	1: w^{1118}(I); *AstC-Gal4, AstC*^{1}>*UAS-dTrpA1*(II)	3 days old; virgin females
	2: w^{1118}(I); *AstC-Gal4, AstC*^{1}>*UAS-dTrpA1, AstC*^{1}(II)	3 days old; virgin females
	3: w^{1118}(I); *AstC-Gal4, AstC*^{1}>+(II)	3 days old; virgin females
	4: w^{1118}(I); *AstC-Gal4, AstC*^{1}>*AstC*^{1}(II)	3 days old; virgin females
Fig. 1e	w^{1118}(I); *AstC-D-Gal4>UAS-Kir2.1*(II)	12~24 HAE; virgin females
	w^{1118}(I); *AstC-D-Gal4>+*(II)	12~24 HAE; virgin females
	w^{1118}(I); *UAS-Kir2.1* >+(II)	12~24 HAE; virgin females
Fig. 1f	w^{1118}(I); *AstC-D-Gal4, AstC*^{1} >+(II)	12 HAE; virgin females
	w^{1118}(I); *AstC*^{1}>*AstC*^{1}(II)	12 HAE; virgin females
	w^{1118}(I); *AstC-D-Gal4, AstC*^{1},*AstC*^{1}(II) > *UAS-AstC* (III)	12 HAE; virgin females
Fig. 2a	1. w^{1118}(I); *AstC-Gal4-DBD* (II), *AstC-D-Gal4-AD* (III) > *UAS-dTrpA1*(II)	3 days old; virgin females
	2. *Dicer2*(I); *AstC-Gal4-DBD* (II), *AstC-D-Gal4-AD* (III) > *UAS-dTrpA1*(II), *UAS-AstC-RNAi* (III)	3 days old; virgin females

	3. *w*^{1118}(I); *AstC-Gal4-DBD*>+ (II), *AstC-D-Gal4-AD*>+ (III)	3 days old; virgin females
	4. *Dicer2* (I); *UAS-dTrpA1*>+(II), *UAS-AstC-RNAi*>+(III)	3 days old; virgin females
	5. *w*^{1118}(I);*UAS-dTrpA1*>+(II)	3 days old; virgin females
Fig. 2b	*w*^{1118}(I); *AstC-Gal4-DBD* (II), *AstC-D-Gal4-AD* (III) > *UAS-mCD8-EGFP*(II)	4 days old; virgin females
Fig. 2d	1. *w*^{1118}(I); *AstC-Gal4-DBD* (II), *AstC-D-Gal4-AD* (III) > *UAS-dTrpA1*(II)	3 days old; virgin females
	2. *w*^{1118}(I);*UAS-dTrpA1*>+(II)	3 days old; virgin females
	3. *w*^{1118}(I); *AstC-Gal4-DBD* (II),*AstC-D-Gal4-AD* (III)>*Otd-Flp*(II),*UAS-stop-dTrpA1*(III)	3 days old; virgin females
	4. *Otd-FLP*>+(II), *UAS-stop-dTrpA1*>+ (III)	3 days old; virgin females
	5. *w*^{1118}(I); *AstC-Gal4-DBD*>+ (II), *AstC-D-Gal4-AD*>+ (III)	3 days old; virgin females
Fig. 2e,f	*w*^{1118}(I); *AstC-Gal4-DBD* (II), *AstC-D-Gal4-AD* (III) > *UAS-mCD8-RFP, LexAop2-mCD8-GFP; nSyb-MKII::nlsLexADBD;UAS-p65AD::CaM*	4 days old; virgin females
Fig. 2g,h	*w*^{1118}(I);*AstC-Gal4-DBD*(II),*AstC-D-Gal4-AD* (III)>*UAS-syt-EGFP*(II),*UAS-denmark* (III)	4 days old; virgin females
Fig. 2i,j	*w*^{1118}(I); *AstC-Gal4-DBD* (II), *AstC-D-Gal4-AD* (III) > *UAS-mCD8-EGFP*(II)	4 days old;virgin females in i
		4 days old; male in j
Fig. 3a	1. *w*^{1118}(I); *AstC-Gal4-DBD* (II), *AstC-D-Gal4-AD* (III) > *UAS-dTrpA1*(II)	1 days old; virgin females
	2. *w*^{1118}(I); *AstC-Gal4-DBD*>+ (II), *AstC-D-Gal4-AD*>+ (III)	1 days old; virgin females
	3. *w*^{1118}(I);*UAS-dTrpA1*>+(II)	1 days old; virgin females
Fig. 3b	1. *w*^{1118}(I); *AstC-Gal4-DBD* (II), *AstC-D-Gal4-AD* (III) > *UAS-dTrpA1*(II)	3 days old; virgin females
	2. *w*^{1118}(I); *AstC-Gal4-DBD* (II), *AstC-D-Gal4-AD* (III) > *UAS-dTrpA1*(II); Methoprene feeding	3 days old; virgin females
	3. *w*^{1118}(I); *AstC-Gal4-DBD*>+ (II), *AstC-D-Gal4-AD*>+ (III)	3 days old; virgin females
	4. *w*^{1118}(I);*UAS-dTrpA1*>+(II)	3 days old; virgin females
Fig. 3c	w*; *AstC-R1-2A-GAL4* (III) > *UAS-mCD8-EGFP*(II)	4 days old; virgin females
Fig. 3d	w*; *AstC-R2-2A-GAL4* (III) > *UAS-mCD8-EGFP*(II)	4 days old; virgin females
Fig. 3e	*Dicer2*(I); *JHAMT-Gal4* (II) >*UAS-AstCR1-RNAi* (III)	12~24 HAE; virgin females
	w^{1118}*(I); UAS-AstCR1-RNAi* >+(III)	12~24 HAE; virgin females
	Dicer2(I); *JHAMT-Gal4* (II) >*UAS-AstCR2-RNAi* (III)	12~24 HAE; virgin females
	w^{1118}*(I); UAS-AstCR2-RNAi* >+(III)	12~24 HAE; virgin females
	Dicer2(I); *JHAMT-Gal4* > +(II)	12~24 HAE; virgin females

Fig. 3f	*Dicer2*(I); *JHAMT-Gal4* (II) >*UAS-AstCR1*(II)	3 days old; virgin females
	w^{1118}*(I); UAS-AstCR1*>+(II)	3 days old; virgin females
	Dicer2(I); *JHAMT-Gal4* (II) >*UAS-AstCR2*(III)	3 days old; virgin females
	w^{1118}*(I); UAS-AstCR2*>+(III)	3 days old; virgin females
	Dicer2(I); *JHAMT-Gal4* (II) >*UAS-AstCR1*(II), *UAS-AstC-R2* (III)	3 days old; virgin females
	w^{1118}*(I); UAS-AstCR1*>+(II), *UAS-AstC-R2*>+ (III)	3 days old; virgin females
	Dicer2(I); *JHAMT-Gal4* > +(II)	3 days old; virgin females
Fig. 4a	w^{1118}(I); *AstC-Gal4-DBD* (II), *AstC-D-Gal4-AD* (III) > *UAS-mCD8-RFP, LexAop2-mCD8-GFP; nSyb-MKII::nlsLexADBD;UAS-p65AD::CaM*	4 days old; virgin females
	Mated with *Conton S* male	4 days old; mated females
	Mated with SP^0>+ male	4 days old; mated females
	Mated with SP^0>$SP\ \Delta^{130}$ male	4 days old; mated females
Fig. 4b	w^{1118}(I); *AstC-Gal4-DBD* (II), *AstC-D-Gal4-AD* (III) > *UAS-dTrpA1*(II)	4 days old; mated females
	w^{1118}(I); *AstC-Gal4-DBD*>+ (II), *AstC-D-Gal4-AD*>+ (III)	4 days old; mated females
	w^{1118}(I);*UAS-dTrpA1*>+(II)	4 days old; mated females
Fig. 4c	*Dicer2*(I); *JHAMT-Gal4* (II) >*UAS-ETHR-RNAi* (III)	4 days old; mated females
	Dicer2(I); *JHAMT-Gal4*> +(II)	4 days old; mated females
	w^{1118}(I); *UAS-ETHR-RNAi*>+ (III)	4 days old; mated females
Fig. 4d	w^{1118}(I); *VT50405-p65-AD*(II), *VT7068-GAL-DBD*(III) > *UAS-dTrpA1* (II)	4 days old; mated females
	w^{1118}(I); *VT50405-p65-AD*>+(II), *VT7068-GAL-DBD*>+(III)	4 days old; mated females
	w^{1118}(I);*UAS-dTrpA1*>+(II)	4 days old; mated females
Fig. 5a	w^{1118}(I); *VT50405-p65-AD*(II), *VT7068-GAL-DBD*(III) > *UAS-Kir2.1* (II)	12~24 HAE; virgin females
	w^{1118}(I); *VT50405-p65-AD*>+(II), *VT7068-GAL-DBD*>+(III)	12~24 HAE; virgin females
	w^{1118}(I);*UAS-Kir2.1*>+(II)	12~24 HAE; virgin females
Fig. 5b	1: w^{1118}(I); *50405-Gal4* (III) > *UAS-dTrpA1*(II)	3 days old; virgin females
	2: w^{1118}(I); *50405-Gal4* (III) >*UAS-dTrpA1, AstC*^{1}(II)	3 days old; virgin females
	3: w^{1118}(I); *50405-Gal4* (III), *AstC*^{1}(II) >*UAS-dTrpA1, AstC*^{1}(II)	3 days old; virgin females
	4: w^{1118}(I); *50405-Gal4* (III) > *UAS-dTrpA1*(II) Methoprene feeding	3 days old; virgin females

	5: w^{1118}(I); *50405-Gal4*> +(III)	3 days old; virgin females
	6: w^{1118}(I); *UAS-dTrpA1*> +(II)	3 days old; virgin females
Fig. 5c	1: w^{1118}(I); *50405-LexA* (III) > *LexAop-dTrpA1*(II)	3 days old; virgin females
	2: w^{1118}(I); *50405-LexA*> + (III)	3 days old; virgin females
	3: w^{1118}(I); *AstC-D-Gal4* (II) >*UAS-Kir2.1*(III)	3 days old; virgin females
	4: w^{1118}(I); *AstC-D-Gal4*>+ (II)	3 days old; virgin females
	5: w^{1118}(I); *50405-LexA* (III), *AstC-D-Gal4* (II) > *LexAop-dTrpA1*(II), *UAS-Kir2.1* (III)	3 days old; virgin females
	6: w^{1118}(I); *LexAop-dTrpA1*> +(II) , *UAS-Kir2.1* >+ (III)	3 days old; virgin females
Fig. 5d	1: w^{1118}(I); *50405-LexA* (III) > *LexAop-Kir2.1*(II)	12~24 HAE; virgin females
	2: w^{1118}(I); *50405-LexA* > +(III)	12~24 HAE; virgin females
	3: w^{1118}(I); *AstC-D-Gal4* (II) >*UAS-dTrpA1*(II)	12~24 HAE; virgin females
	4: w^{1118}(I); *AstC-D-Gal4* >+(II)	12~24 HAE; virgin females
	5: w^{1118}(I); *50405-LexA* (III), *AstC-D-Gal4* (II) > *LexAop-Kir2.1*(II), *UAS-dTrpA1* (II)	12~24 HAE; virgin females
	6: w^{1118}(I); *LexAop-kir2.1*, *UAS-dTrpA1* >+(II)	12~24 HAE; virgin females
Fig. 5e	w^{1118}(I); *VT50405-p65-AD*(II), *VT7068-GAL-DBD*(III) > *UAS-mCD8-RFP, LexAop2-mCD8-GFP; nSyb-MKII::nlsLexADBD;UAS-p65AD::CaM*	0~3 DAE: virgin females
Fig. S1a	w^{1118}(I); *AstC-Gal4, AstC*^{1}> +(II)	12 HAE; virgin females
	w^{1118}(I); *AstC*^{1}>*AstC*^{1}(II)	12 HAE; virgin females
	w^{1118}(I); *AstC-Gal4, AstC*^{1},*AstC*^{1}(II) > *UAS-AstC* (III)	12 HAE; virgin females
Fig. S1b	w^{1118}(I); *AstC-Gal4, AstC*^{1}> +(II)	0~120 HAE; virgin females
	w^{1118}(I); *AstC*^{1}>*AstC*^{1}(II)	0~120 HAE; virgin females
Fig. S1c	w^{1118}(I); *AstC-Gal4, AstC*^{1}> +(II)	Eggs of 4 days old mated females
	w^{1118}(I); *AstC*^{1}>*AstC*^{1}(II)	Eggs of 4 days old mated females
Fig. S1d	w^{1118}(I); *AstC-CNMa-Gal4, AstC*^{1}>+(II)	12 HAE; virgin females
	w^{1118}(I); *AstC*^{1}>*AstC*^{1}(II)	12 HAE; virgin females
	w^{1118}(I); *AstC-CNMa-Gal4, AstC*^{1},*AstC*^{1}(II) > *UAS-AstC* (III)	12 HAE; virgin females
Fig. S1e	w^{1118}(I); *AstC-D-Gal4/UAS-dTrpA1*(II)	3 days old; virgin females
	w^{1118}(I); *AstC-D-Gal4*>+(II)	3 days old; virgin females
	w^{1118}(I); *UAS-dTrpA1*>+II)	3 days old; virgin females

Fig. S1f	w^{1118}(I); *AstC-D-Gal4>UAS-Kir2.1*(II)	3 days old; virgin females
	w^{1118}(I); *AstC-D-Gal4*>+(II)	3 days old; virgin females
	w^{1118}(I); *UAS-Kir2.1*>+(II)	3 days old; virgin females
Fig. S1g	w^{1118}(I); *AstC-D-Gal4>UAS-Shi* ^{ts}(II)	12~24 HAE; virgin females
	w^{1118}(I); *AstC-D-Gal4*>+(II)	12~24 HAE; virgin females
	w^{1118}(I); *UAS-Shit* ^{ts}>+(II)	12~24 HAE; virgin females
Fig. S2a	w^{1118}(I); *AstC-Gal4*(II)> *UAS-mCD8-EGFP*(II)	4 days old; virgin females
Fig. S2b	w^{1118}(I); *AstC-D-Gal4*(II)> *UAS-mCD8-EGFP*(II)	4 days old; virgin females
Fig. S2c	w^{1118}(I); *AstC-Gal4-DBD* (II), *AstC-D-Gal4-AD* (III) > *UAS-mCD8-EGFP*(II)	4 days old; virgin females
Fig. S2d	*Dicer2*(I); *AstC-Gal4-DBD* (II), *AstC-D-Gal4-AD* (III) > *UAS-dTrpA1*(II), *UAS-AstC-RNAi* (III)	4 days old; virgin females
Fig. S2e	*Dicer2* (I); *UAS-dTrpA1*>+(II), *UAS-AstC-RNAi*>+(III)	4 days old; virgin females
Fig. S2f	w^{1118}(I); *AstC-Gal4-DBD* (II), *AstC-D-Gal4-AD* (III) > *Otd-Flp*(II), *UAS-stop-EGFP*(III)	4 days old; virgin females
Fig. S2g	w^{1118}(I); *AstC-Gal4-DBD* (II), *AstC-D-Gal4-AD* (III) > *UAS-mCD8-RFP, LexAop2-mCD8-GFP; nSyb-MKII::nlsLexADBD;UAS-p65AD::CaM*	4 days old; virgin females
		4 days old; virgin females
Fig. S2h	w^{1118}(I); *AstC-Gal4-DBD* (II), *AstC-D-Gal4-AD* (III) > *UAS-mCD8-RFP, LexAop2-mCD8-GFP; nSyb-MKII::nlsLexADBD;UAS-p65AD::CaM*	(-24~72) HAE: virgin females
Fig. S3a	1.w^{1118} ,Stage 8 pupae	Stage 8 pupae
	2.w^{1118} ,Day 1 adult	1 day old; virgin females
	3.w^{1118}(I); *AstC-D-Gal4>UAS-dTrpA1*(II) Day 1 adult	1 day old; virgin females
Fig. S3b	1.w^{1118} ,Stage 8 pupae	Stage 8 pupae
	2.w^{1118} ,Day 1 adult	1 day old; virgin females
	3.w^{1118}(I); *UAS-dTrpA1*>+(II)	1 day old; virgin females
	4.w^{1118}(I); *AstC-D-Gal4*>+(II)	1 day old; virgin females
	5.w^{1118}(I); *AstC-D-Gal4>UAS-dTrpA1*(II)	1 day old; virgin females
Fig. S4a	1. w^{1118}(I); $AstC^{1}$>$AstC^{1}$(II)	1 day old; virgin females
	2. w^{1118}(I); $AstC^{1}$>+(II)	1 day old; virgin females
Fig. S4b	1. w^{1118}(I); $AstC^{1}$>$AstC^{1}$(II)	4 days old; virgin females

	2. *w*^{1118}(I); *AstC*^{1}>+(II)	4 days old; virgin females
Fig. S4c	1. *w*^{1118}(I); *AstC-Gal4-DBD* (II), *AstC-D-Gal4-AD* (III) > *UAS-Kir2.1* (II)	4 days old; virgin females
	2. w^{1118}(I); *AstC-Gal4-DBD*>+(II), *AstC-D-Gal4-AD*>+ (III)	4 days old; virgin females
	3. *w*^{1118}(I);*UAS-Kir2.1*>+(II)	4 days old; virgin females
Fig.S4d	*w*^{1118}(I); *AstC-Gal4-DBD* (II), *AstC-D-Gal4-AD* (III) > *UAS-mCD8-RFP, LexAop2-mCD8-GFP; nSyb-MKII::nlsLexADBD;UAS-p65AD::CaM*	1 day old; virgin females
		1 day old; virgin females
Fig S5a	*w*^{1118}(I); *AstC-Gal4-DBD* (II), *AstC-D-Gal4-AD* (III) > *UAS-myr-EGFP*(II)	4 days old; virgin females
Fig S5b	*JHAMT-Gal4* (II) >*UAS-mCD8-GFP*(II)	4 days old; virgin females
Fig. S6a	1.*w*^{1118}(I); *UAS-dTrpA1*>+(II)	3 days old; virgin females
	2.*w*^{1118}(I); *AstC-D-Gal4*>+(II)	3 days old; virgin females
	3.*w*^{1118}(I); *AstC-D-Gal4>UAS-dTrpA1*(II)	3 days old; virgin females
Fig. S6b	1.*w*^{1118}(I); *UAS-NaChBac*>+(II)	5 days old; virgin females
	2.*w*^{1118}(I); *AstC-D-Gal4*(II) >+	5 days old; virgin females
	3.*w*^{1118}(I); *AstC-D-Gal4>UAS-NaChBac*(II)	5 days old; virgin females
Fig. S7a~h	*w*^{1118}	
	Mated with *Conton S*	4 days old; mated females
Fig. S7g	*w*^{1118}	4 days old; mated females
	Mated with *Conton S*	4 days old; mated females
	Mated with *SP*^{0}>*SPΔ*^{130}	4 days old; mated females
Fig. S8a	*w*^{1118}*(I); AstC-Gal4-DBD* (II)*, AstC-D-Gal4-AD* (III) >*UAS-dTrpA1*(II)	5 days old; mated females
	w^{1118}(I); *AstC-Gal4-DBD*>+(II), *AstC-D-Gal4-AD*>+ (III)	5 days old; mated females
	w^{1118}(I); *UAS-dTrpA1*>+(II)	5 days old; mated females
Fig. S8b	*w*^{1118}*(I); AstC-Gal4-DBD* (II)*, AstC-D-Gal4-AD* (III) >*UAS-Shi*^{ts}(II)	4 days old; virgin females
	w^{1118}(I); *AstC-Gal4-DBD*>+(II), *AstC-D-Gal4-AD*>+ (III)	4 days old; virgin females
	w^{1118}(I); *UAS-Shit*^{ts}>+(II)	4 days old; virgin females

Fig. S9a	*w*^{1118}(I); *VT50405-p65-AD*(II), *VT7068-GAL-DBD*(III) > *UAS-syt-EGFP* (II)	4 days old; virgin females
Fig. S9b	*w*^{1118}(I); *VT50405-p65-AD*(II), *VT7068-GAL-DBD*(III) > *P{UAS-myrGFP.QUAS-mtdTomato-3xHA}su(Hw)*(X); *P{trans-Tango}* (II)	4 days old; virgin females
Fig. S10	*w*^{1118}(I); AstC-D-Gal4 (II), *50405-LexA* (III)> *20XUAS-IVS-GCaMP6m* (II), *13XLexAop2-IVS0csChrimson.mVenus* (III)	3 days old; virgin females
