# Supplementary material for: The insect somatostatin pathway gates vitellogenesis progression during reproductive maturation and the post-mating response
Source: Nat Commun. 2022 Feb 18;13:969. doi: 10.1038/s41467-022-28592-2 (PMC8857180; doi:10.1038/s41467-022-28592-2)
Supplement: Supplementary file 3 — Reporting Summary [file 41467_2022_28592_MOESM3_ESM.pdf]

nature portfolio

Corresponding author(s): Young-Joon Kim

Last updated by author(s): Jan 21, 2022

Reporting Summary

Nature Portfolio wishes to improve the reproducibility of the work that we publish. This form provides structure for consistency and transparency in reporting. For further information on Nature Portfolio policies, see our Editorial Policies and the Editorial Policy Checklist.

Statistics

For all statistical analyses, confirm that the following items are present in the figure legend, table legend, main text, or Methods section.

n/a	Confirmed	
☐	☒	The exact sample size (*n*) for each experimental group/condition, given as a discrete number and unit of measurement
☐	☒	A statement on whether measurements were taken from distinct samples or whether the same sample was measured repeatedly
☐	☒	The statistical test(s) used AND whether they are one- or two-sided<br>*Only common tests should be described solely by name; describe more complex techniques in the Methods section.*
☒	☐	A description of all covariates tested
☐	☒	A description of any assumptions or corrections, such as tests of normality and adjustment for multiple comparisons
☐	☒	A full description of the statistical parameters including central tendency (e.g. means) or other basic estimates (e.g. regression coefficient) AND variation (e.g. standard deviation) or associated estimates of uncertainty (e.g. confidence intervals)
☐	☒	For null hypothesis testing, the test statistic (e.g. *F*, *t*, *r*) with confidence intervals, effect sizes, degrees of freedom and *P* value noted<br>*Give P values as exact values whenever suitable.*
☒	☐	For Bayesian analysis, information on the choice of priors and Markov chain Monte Carlo settings
☒	☐	For hierarchical and complex designs, identification of the appropriate level for tests and full reporting of outcomes
☒	☐	Estimates of effect sizes (e.g. Cohen's *d*, Pearson's *r*), indicating how they were calculated

Our web collection on statistics for biologists contains articles on many of the points above.

Software and code

Policy information about availability of computer code

Data collection	Confocal data: LSM700/Axiovert 200M (Zeiss) with Zeiss Zen 2009 software (version 6,0,0,303)<br>Wing size measurement: LEICA EZ4E with LAS V4.10 softerware<br>Feeding behaviors: Fly Liquid-Food Interaction Counter, Sable systems<br>RT-qPCR: StepOnePlus Real-Time PCR system, Applied Biosystems
Data analysis	Fiji - ImageJ (v1.52n)<br>Prism5 for windows (v7.00) - GraphPad Software Inc.<br>MATLAB R2021a (9.10.0.1649659)<br>StepOne Software v2.3

For manuscripts utilizing custom algorithms or software that are central to the research but not yet described in published literature, software must be made available to editors and reviewers. We strongly encourage code deposition in a community repository (e.g. GitHub). See the Nature Portfolio guidelines for submitting code & software for further information.

Data

Policy information about availability of data

All manuscripts must include a data availability statement. This statement should provide the following information, where applicable:

- Accession codes, unique identifiers, or web links for publicly available datasets
- A description of any restrictions on data availability
- For clinical datasets or third party data, please ensure that the statement adheres to our policy

The source data underlying Figs. 1a, c-f, 2a, d, 3a, b, e, f, 4a-d, 5a-e, and Supplementary Figs 1b-g, 2h, 3a, b, 4a-d, 6a, b, 7a-h, and 8a, b are provided as a Source

data file.
Additional raw data comprising confocal micrograph stacks and electrophysiological recordings are available from http://doi.org/10.6084/m9.figshare.18737786.
A MATLAB script used for FRIC analysis is available from https://github.com/daeyeonkim/FLIC.

Field-specific reporting

Please select the one below that is the best fit for your research. If you are not sure, read the appropriate sections before making your selection.

☒ Life sciences ☐ Behavioural & social sciences ☐ Ecological, evolutionary & environmental sciences

For a reference copy of the document with all sections, see nature.com/documents/nr-reporting-summary-flat.pdf

Life sciences study design

All studies must disclose on these points even when the disclosure is negative.

Sample size	Sample sizes were determined via reference to prior studies using equivalent techniques or standard in our lab.<br>*Number of oocytes: the standard in our lab. Zhang, C. et al. The neuropeptide allatostatin C from clock-associated DN1p neurons generates the circadian rhythm for oogenesis. Proc. Natl. Acad. Sci. U. S. A. 118, (2021).<br>*Egg Staging: Jia, D., Xu, Q., Xie, Q., Mio, W. & Deng, W. M. Automatic stage identification of Drosophila egg chamber based on DAPI images. Sci. Rep. 6, 1–12 (2016). Meiselman, M. et al. Endocrine network essential for reproductive success in Drosophila melanogaster. Proc. Natl. Acad. Sci. U. S. A. 114, E3849–E3858 (2017).<br>*RT-qPCR: 6 samples, each containing 5 animals, the standard in our lab.<br>*TRIC activity: Jang, Y. H., Chae, H. S. & Kim, Y. J. Female-specific myoinhibitory peptide neurons regulate mating receptivity in Drosophila melanogaster. Nat. Commun. 8, 1–12 (2017).<br>*JHIII measurement: Meiselman, M. et al. Endocrine network essential for reproductive success in Drosophila melanogaster. Proc. Natl. Acad. Sci. U. S. A. 114, E3849–E3858 (2017).<br>*Feeding behavior (FLIC): refer to a recent preprint paper "Kubrak, O. et al. The gut hormone Allatostatin C regulates food intake and metabolic homeostasis under nutrient stress. Preprint at bioRxiv 2020.12.05.412874 (2020).", which confirmed feeding behaviors of AstC mutant flies via FLIC.<br>*Dilp2 intensity: the standard in our lab. Zhang, C. et al. The neuropeptide allatostatin C from clock-associated DN1p neurons generates the circadian rhythm for oogenesis. Proc. Natl. Acad. Sci. U. S. A. 118, (2021).<br>*Wing size measurement: the standard in our lab. Zhang, C. et al. The neuropeptide allatostatin C from clock-associated DN1p neurons generates the circadian rhythm for oogenesis. Proc. Natl. Acad. Sci. U. S. A. 118, (2021).<br>*Electrophysiology: according to typical sample sizes in the field.
Data exclusions	No data were excluded.
Replication	All experiments were replicated at least once independently. The repeating experiment was performed on different days using flies from different batches or independent genetic crosses. All attempts at replication were successful.
Randomization	Flies that carry same genotype from either test or control groups were randomly chosen for experiments. For each experiment, the test and control group of flies were cultured via the same way in the vials, collected, treated, and tested at the same time that has been indicated.
Blinding	Investigators were not blinded to group allocation during data collection and analysis, as these (i.e., oocyte, egg counting, and immunohistochemistry, TRIC measurement, and RT-qPCR) were nonsubjective. JH measurement and electrophysiology had been performed in collaborative labs, reducing the risk of bias.

Reporting for specific materials, systems and methods

We require information from authors about some types of materials, experimental systems and methods used in many studies. Here, indicate whether each material, system or method listed is relevant to your study. If you are not sure if a list item applies to your research, read the appropriate section before selecting a response.

Materials & experimental systems

n/a	Involved in the study	
☐	☒	Antibodies
☒	☐	Eukaryotic cell lines
☒	☐	Palaeontology and archaeology
☐	☒	Animals and other organisms
☒	☐	Human research participants
☒	☐	Clinical data
☒	☐	Dual use research of concern

Methods

n/a	Involved in the study	
☒	☐	ChIP-seq
☒	☐	Flow cytometry
☒	☐	MRI-based neuroimaging

Antibodies

Antibodies used	Primary antibodies:<br>*Rabbit anti-GFP (Invitrogen, A11122), (1:1000 dilution);<br>*Mouse anti-GFP (Invitrogen, A11120) (1:1000 dilution);<br>*Rabbit anti-DsRed (Clontech, 632496) (1:1000 dilution);<br>*Rabbit anti-AstC (A gift from Dušan Žitňan, Slovak Academy of Sciences) (1:1000 dilution);<br>*Rat anti-HA (Roche, 11867423001) (1:100 dilution);<br>*Rabbit anti-JHAMT (a gift from Ryusuke Niwa, University of Tsukuba) (1:1000 dilution);<br>*Rabbit anti-Dilp2 (a gift from Yu Kweon from Korea Research Institute of Bioscience and Biotechnology) (1:1000 dilution);<br>*Mouse anti-nc82 (Developmental Studies Hybridoma Bank, Bruchpilot) (1:50 dilution).<br>Secondary antibodies:<br>*Alexa Fluor 488-labeled goat anti-rabbit IgG (1:1000 dilution; Invitrogen, A11008)<br>*Alexa Fluor 488-labeled goat anti-mouse IgG (1:1000 dilution; Invitrogen, A11001)<br>*Alexa Fluor 568 goat anti-rabbit IgG (1:1000 dilution; Invitrogen, A11011)<br>*Alexa Fluor 568 goat anti-mouse IgG (1:1000 dilution; Invitrogen, A11004)<br>*Alexa Fluor 633 goat anti-rat IgG (1:500 dilution; Invitrogen, A21094)
Validation	Validation of primary antibodies:<br>*Rabbit anti-GFP (Invitrogen, A11122): immunohistochemistry, (Thermofisher Sci website, under product specification).<br>*Mouse anti-GFP (Invitrogen, A11120): immunohistochemistry, (Thermofisher Sci website, under product specification).<br>*Rabbit anti-DsRed (Clontech, 632496): Jang, Y. H., Chae, H. S. & Kim, Y. J. Female-specific myoinhibitory peptide neurons regulate mating receptivity in Drosophila melanogaster. Nat. Commun. 8, 1–12 (2017).<br>*Rabbit anti-AstC: Žitňan, D., Kingan,T.G., Kramer,S.J., Beckage, N.E.. Accumulation of neuropeptides in the cerebral neurosecretory system of Manduca sexta larvae parasitized by the braconid wasp Cotesia congregata. J. Comp. Neurol. 356, 83–100 (1995). Zhang, C. et al. The neuropeptide allatostatin C from clock-associated DN1p neurons generates the circadian rhythm for oogenesis. Proc. Natl. Acad. Sci. U. S. A. 118, (2021).<br>*Rat anti-HA (Roche, 11867423001): Hampel, S. et al. Drosophila Brainbow: A recombinase-based fluorescence labeling technique to subdivide neural expression patterns. Nat. Methods 8, 253–259 (2011).<br>*Rabbit anti-JHAMT: Niwa, R. et al. Juvenile hormone acid O-methyltransferase in Drosophila melanogaster. Insect Biochem. Mol. Biol. 38, 714–720 (2008).<br>*Rabbit anti-Dilp2: Kwak S.J. et al., Drosophila adiponectin receptor in insulin producing cells regulates glucose and lipid metabolism by controlling insulin secretion. PLoS One 8, e68641 (2013). Zhang, C. et al. The neuropeptide allatostatin C from clock-associated DN1p neurons generates the circadian rhythm for oogenesis. Proc. Natl. Acad. Sci. U. S. A. 118, (2021).<br>*Mouse anti-nc82(Developmental Studies Hybridoma Bank, Bruchpilo): immunohistochemistry (DSHB website, under catalog fields).

Animals and other organisms

Policy information about studies involving animals; ARRIVE guidelines recommended for reporting animal research

Laboratory animals	Only Drosophila melanogaster (invertebrate) was used in this study.<br>Flies were raised at 25°C and 60% humidity under a 12 h:12 h light:dark cycle on standard fly media. The stocks used in this study were indicated in Methods (Fly stocks).<br>Ages of test animals were indicated in experimental protocol shown in Figures, Supplemental Table 2, or Methods.<br>A full list of detailed genotypes, age, sex and mating status of flies examined in this study is provided in Supplementary Table 2.
Wild animals	This study did not involve wild animals.
Field-collected samples	This study did not involve samples collected from the field.
Ethics oversight	This study did not require an ethical approval.

Note that full information on the approval of the study protocol must also be provided in the manuscript.
